# Phase-specific alpha tACS over the dlPFC does not enhance cognitive reappraisal or autonomic responses: A sham-controlled crossover study

**DOI:** 10.3934/Neuroscience.2026022

**Published:** 2026-08-26

**Authors:** Catarina Gomes Coelho, Jorge Leite, Gabriela Gonzaga, Paulo Machado, Sandra Carvalho

**Affiliations:** 1 Psychology Research Centre (CIPsi), Department of Basic Psychology, School of Psychology, University of Minho, Braga, Portugal; 2 Psychology Research Centre (CIPsi), Department of Applied Psychology, School of Psychology, University of Minho, Braga, Portugal; 3 CINTESIS@RISE, CINTESIS.UPT, Portucalense University, Porto, Portugal

**Keywords:** ER, cognitive reappraisal, tACS, dlPFC, alpha oscillations, autonomic physiology, phase-specific

## Abstract

**Background:**

Cognitive reappraisal (CR) relies on prefrontal top-down control over limbic systems, with the dorsolateral prefrontal cortex (dlPFC) playing a central regulatory role. Alpha-frequency transcranial alternating current stimulation (α-tACS) can modulate cortical oscillations; yet, its causal contribution to emotion regulation (ER) remains unclear.

**Objective:**

In this study, we investigated whether in-phase or anti-phase alpha-tACS influenced subjective and physiological responses during cognitive reappraisal of negative emotional stimuli.

**Methods:**

Forty-two healthy young adults completed three counterbalanced sessions (in-phase, anti-phase, sham) in a single-blind, sham-controlled crossover design. During α-tACS or sham, participants applied three CR strategies (positive, fictional, distancing) while viewing negative and neutral film clips. Valence, arousal, heart rate (HR), skin conductance (SC), respiratory rate (RR), and pre/post-session affect (PANAS) were recorded.

**Results:**

The Emotional Movie Database (EMDB) emotional stimuli elicited the expected subjective and autonomic responses, confirming that the task engaged emotional-reactivity processes. Participants also showed a typical habituation pattern in physiological measures across blocks. However, neither in-phase nor anti-phase alpha-tACS was associated with measurable changes in subjective emotional ratings or autonomic responses relative to sham under the experimental conditions.

**Conclusion:**

This study provides the first evaluation of phase-specific alpha-tACS during cognitive reappraisal using film-based stimuli. Under the experimental conditions, no measurable effects of in-phase or anti-phase stimulation on subjective or autonomic measures of ER were observed. Future studies incorporating individualized stimulation frequencies, multi-session protocols, closed-loop stimulation, and concurrent electrophysiological recordings may help clarify the conditions under which phase-specific tACS influences ER.

## 1. Introduction

Emotion regulation (ER) is widely recognized as a core transdiagnostic process with substantial implications for mental health and resilience. When regulation is weak or inconsistent, individuals are more likely to develop psychiatric disorders, exhibit heightened stress reactivity, and experience difficulties in daily adaptation. These vulnerabilities extend beyond clinical populations: Non-clinical individuals with persistent ER difficulties often show reduced flexibility and poorer adjustment in academic, occupational, and social contexts. Consequently, strengthening ER capacities has become a priority target for intervention and prevention efforts aimed at promoting psychological well-being [1],[2].

Among the available strategies, cognitive reappraisal (CR) consistently emerges as one of the most effective. CR involves reinterpreting the meaning of an emotional stimulus in ways that alter its impact, such as construing it more positively, adopting psychological distance, or framing it as fictional. More specifically, positive reappraisal refers to interpreting the situation in a more positive or beneficial way, while distancing involves adopting an external observer's perspective to reduce emotional involvement, and fictional reappraisal consists of interpreting the emotional event as unreal or fictional, thus diminishing its affective impact. Because CR acts early in the emotion-generation sequence, it is considered an antecedent-focused strategy that can redirect the emotional trajectory before responses fully unfold [3].

From a neuroimaging perspective, CR relies on top-down regulatory mechanisms supported by prefrontal cortical (PFC) regions, mostly the dorsolateral prefrontal cortex (dlPFC), ventrolateral and ventromedial prefrontal regions, and the dorsal anterior cingulate cortex, which modulate limbic reactivity, particularly amygdala responses to emotionally salient stimuli [4]–[8]. Successful reappraisal requires sustained goal maintenance, attentional control, and the inhibition of prepotent affective responses, processes that are reflected in oscillatory dynamics within fronto-cortical networks. Alpha-band oscillations (8–12 Hz) are widely interpreted as markers of functional inhibition and attentional gating, regulating cortical excitability and the allocation of processing resources [9],[10]. In emotional contexts, alpha activity has been associated with the modulation of affective processing and the implementation of regulatory strategies [11].

Converging evidence implicates frontal alpha asymmetry (FAA) in ER. Greater relative left-frontal alpha power has been associated with the use of CR [12]–[14]. FAA also appears more pronounced during active regulation compared with resting states, suggesting that it reflects dynamic regulatory engagement rather than a static trait [15]. A stronger left-FAA pattern has been linked to more effective automatic regulation during stress [16]. However, these findings are predominantly correlational, leaving unresolved whether modulating oscillatory activity can causally influence ER.

Non-invasive brain stimulation (NIBS) provides a way to address this gap by modulating cortical excitability in targeted networks and testing causal hypotheses about brain-behavior relationships. NIBS protocols appear to be able to model alpha and theta activity, particularly in frontal networks [17], and may influence neural systems involved in mood processing. Transcranial magnetic stimulation (TMS) and transcranial direct current stimulation (tDCS) have been widely used in the treatment of mood disorders. Moreover, transcranial alternating current stimulation (tACS) has emerged as a distinctive approach capable of modulating intra- and inter-regional oscillatory activity. By delivering low-intensity, frequency-specific sinusoidal currents, tACS is thought to entrain endogenous neural rhythms to the externally applied frequency, promoting phase synchronization and amplifying cortical oscillations near the stimulation frequency [18]–[20].

Clinical and experimental studies provide growing evidence that tACS may influence cognitive and emotional functions, although the available findings have been obtained using substantially different populations, experimental paradigms, stimulation protocols, and outcome measures. Studies in clinical populations have primarily focused on symptom improvement, whereas studies in healthy participants have examined cognitive and emotional processing. Given these methodological differences, direct comparisons across studies remain challenging. For example, alpha- and theta-frequency protocols have been associated with improvements in emotional and cognitive symptoms in Alzheimer's disease [21], schizophrenia [22] and obsessive–compulsive disorder [23]. A study by Liu et al. [24] reported that alpha-tACS enhanced attentional processes involved in emotion processing, as reflected by increased alpha power and P200 amplitude. McAleer et al. [25] showed that theta-tACS reduced anxiety and depression symptoms while lowering valence and arousal ratings. In a subsequent study, individuals with internalizing psychopathologies exhibited stronger heart-rate modulation during reappraisal when stimulated with tACS compared with tDCS [26]. However, results are not fully convergent, especially for autonomic indices; several studies have reported minimal or absent effects of tACS on measures such as skin conductance [27],[28].

Taken together, the available evidence indicates that the effects of tACS on ER remain heterogeneous. This variability is likely explained by the combined influence of participant characteristics, experimental paradigms, outcome measures, and stimulation parameters rather than by stimulation parameters alone. Among these factors, the phase relationship between stimulation sites remains comparatively underexplored, despite its potential importance for large-scale network communication and interhemispheric synchronization. Unlike other forms of non-invasive brain stimulation, tACS enables not only frequency-specific entrainment but also precise manipulation of phase relationships between cortical regions. Based on previous theoretical and experimental work, in-phase (0°) stimulation is expected to promote synchronization of neural oscillations across stimulated regions, whereas anti-phase (180°) stimulation may reduce or disrupt functional coupling. Comparing these stimulation modes therefore provides a direct experimental framework for testing whether synchrony and desynchrony within prefrontal networks differentially influence ER.

The alpha frequency band (8–12 Hz) was selected because frontal alpha oscillations have consistently been implicated in attentional control, inhibitory processing, and ER. In particular, frontal alpha asymmetry has been associated with cognitive reappraisal, while experimental studies suggest that alpha-tACS can modulate attentional processes relevant to emotional regulation. Together, these findings provide a theoretical rationale for investigating whether phase-specific alpha-tACS can causally influence the neural mechanisms supporting cognitive reappraisal.

Building on this rationale, the dlPFC represents a compelling target for alpha-tACS because of its central role in top-down ER and the involvement of alpha oscillations in attentional gating and inhibitory control. By targeting alpha-band activity, stimulation may influence the functional coordination of prefrontal networks that support goal maintenance and suppression of prepotent affective responses. The use of dynamic and ecologically valid stimuli, such as emotional film clips, increases regulatory demands relative to static images and enables assessment of subjective and autonomic indices of regulation under sustained emotional engagement. Testing these mechanisms in healthy individuals permits evaluation of causal oscillatory contributions to reappraisal-related processes without the confounding influence of disorder-related baseline network alterations [8],[9].

In this study, we examined whether alpha-band tACS applied over the dlPFC, combined with CR strategies (positive reappraisal, fictional reappraisal, and distancing), modulates emotional responses to negative film clips. We tested: (1) Whether subjective ratings of valence and arousal differed across in-phase (0°), anti-phase (180°), and sham stimulation; (2) whether active stimulation influenced physiological markers, including heart rate, skin conductance, and respiratory rate; and (3) whether stimulation influenced broader affective self-report measures, including the Positive and Negative Affect Schedule (PANAS) and Visual Analogue Scales (VAS).

We hypothesized that in-phase stimulation would strengthen interhemispheric synchronization and facilitate regulatory engagement, whereas anti-phase stimulation would interfere with coordinated prefrontal dynamics, resulting in differential modulation of subjective and physiological indices of ER compared with sham.

## 2. Materials and methods

### 2.1. Experimental design

The experiment followed a randomized, single-blind, sham-controlled, within-subject crossover design. Each participant completed three sessions: two with active tACS (in-phase and anti-phase) and one with sham stimulation. Session order was randomized using a computer-generated list and counterbalanced across participants in blocks of 2 and 4 to avoid order effects (https://www.randomizer.org/). Sessions were separated by at least 48 hours, with all three completed over a minimum of six days to minimize potential carryover effects [29]. All procedures were approved by the Ethics Committee for Research in Social and Human Sciences at the University of Minho (CEICSH 202/2024). Data collection was conducted at the Neuroscience Laboratory of Universidade Portucalense, Infante D. Henrique in Porto, Portugal.

During each session, participants performed a negative emotion induction task using film clips from the Emotional Movie Database (EMDB). Concurrently, they were instructed to apply previously trained CR strategies while receiving tACS, either the active or sham condition. Self-report measures included valence and arousal ratings, as well as the selection of the emotion that better represented their emotional experience during each film. Psychophysiological responses (heart rate, skin conductance, and respiratory rate) were continuously monitored throughout the task. Additionally, participants' perceptions of their affective and physiological states were assessed before and after each session using the PANAS and VAS (Figure 1A and 1B).

**Figure 1.**
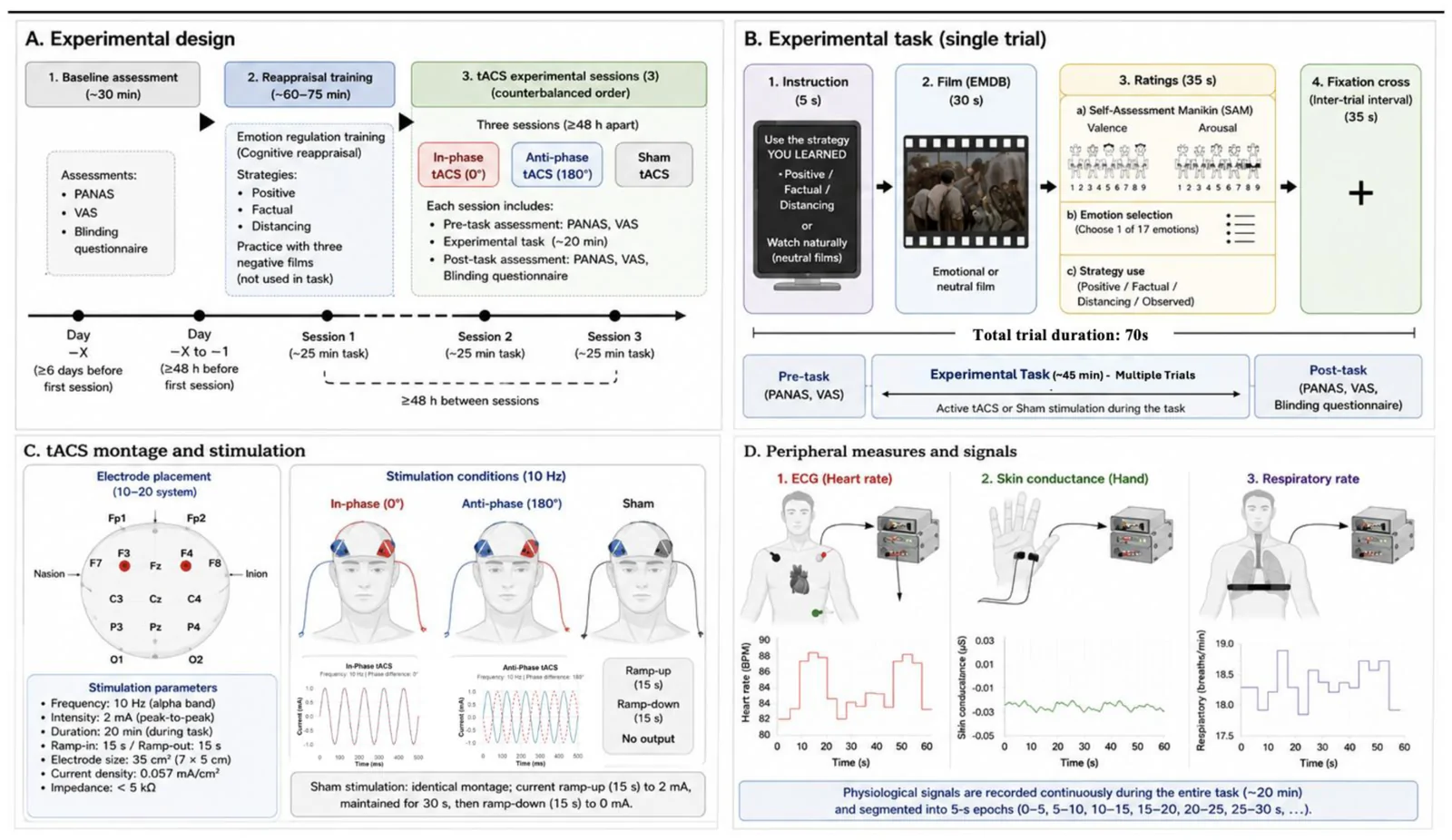
Overview of the experimental protocol, tACS montage, and physiological recordings. (A) Experimental design. Following eligibility screening, baseline assessment, and cognitive reappraisal training, participants completed three counterbalanced experimental sessions (in-phase, anti-phase, and sham tACS), each including pre- and post-task assessments. (B) Experimental task. Each trial consisted of a 5 s instruction period, a 30 s emotional or neutral film clip from the Emotional Movie Database (EMDB), a 35 s evaluation period, including valence and arousal ratings, using the Self-Assessment Manikin (SAM) and emotion selection, and a fixation cross. Positive and Negative Affect Schedule (PANAS) and Visual Analogue Scale (VAS) assessments were administered before and after each experimental session. (C) tACS montage and stimulation conditions. Stimulation was delivered bilaterally over the dorsolateral prefrontal cortex (F3/F4; international 10–20 EEG system) at 10 Hz using in-phase (0°), anti-phase (180°), or sham stimulation. (D) Physiological recordings. Heart rate (ECG), skin conductance (hand electrodes), and respiratory rate (respiratory belt) were continuously recorded throughout the experimental task.

### 2.2. Participants

To ensure linguistic and cultural homogeneity, as well as the capacity to comprehend and follow the study procedures, participants had to be 18 years or older, native Portuguese speakers, and permanent residents of Portugal.

Exclusion criteria were established to minimize risks associated with tACS and to control for potential confounding variables. Participants were excluded if they had any known contraindications for tACS, such as metallic implants in the head (excluding the oral cavity) or implanted medical devices (e.g., pacemakers or neurostimulators). Additional exclusion criteria included a self-reported history of neurological disorders (e.g., epilepsy), current use of neurological or psychiatric medication, use of illicit psychotropic substances, and left-handedness. Handedness was assessed using the Edinburgh Handedness Inventory, with scores below 70 indicating exclusion. The restriction to right-handed individuals aimed to reduce variability in cortical organization and hemispheric lateralization, which could influence the effects of brain stimulation [30].

Although 143 individuals completed the eligibility questionnaire, 43 were deemed ineligible for participation due to exclusion criteria (e.g., neurological disorders, left-handedness). From this group, 45 participants began the experimental sessions. Two participants dropped out during the study, and one participant completed the same task version twice, resulting in a final sample of 42 participants who completed all three sessions.

### 2.3. Measures and instruments

Eligibility was assessed using a 14-item sociodemographic and clinical questionnaire designed to characterize the sample and identify variables relevant to neuromodulation safety and emotional processing. Participants also completed a 16-item screening form covering potential contraindications for tACS, including personal and family history of seizures, neurological disorders, severe head injury, metal implants, implanted medical devices, pregnancy status, current medication use, and previous exposure or adverse reactions to tACS. Handedness was evaluated with the Portuguese version of the Edinburgh Handedness Inventory [31],[32] and only individuals scoring ≥70 were included to reduce variability in hemispheric lateralization.

To characterize participants' cognitive-emotional profiles at baseline, a set of validated Portuguese adaptations of standardized self-report measures was administered. These included the Difficulties in ER Scale – Short Form (DERS-18 [33]), assessing ER difficulties; the ER Questionnaire (ERQ-10 [3]), measuring habitual use of cognitive reappraisal and expressive suppression; The Cognitive Emotion Regulation Questionnaire (CERQ-36; [34],[35]) assessing cognitive regulation strategies following negative events; the Depression Anxiety Stress Scale (DASS-21 [36]); and the Marlowe–Crowne Social Desirability Scale (MCSDS-33 [37]). Complete descriptions and psychometric properties of these measures are provided in the Supplementary Material – Description S1.

### 2.4. ER training session

Before the experimental sessions, all participants completed a standardized cognitive reappraisal (CR) training session designed to ensure a consistent understanding and implementation of the ER strategies used throughout the experiment. The training introduced three CR strategies, positive reappraisal, fictional reappraisal, and distancing, through brief theoretical explanations, standardized verbal instructions, and supervised practice. Positive reappraisal involved reinterpreting the emotional meaning of a situation in a more positive or beneficial way. Fictional reappraisal involved viewing the events as unreal or as part of a fictional narrative to reduce their emotional impact. Distancing involved adopting the perspective of a detached observer to decrease personal emotional involvement in the scene.

Following the instructions, participants practiced each strategy while viewing three negative film clips that were not included in the experimental task, enabling them to rehearse the procedures under conditions closely resembling those of the stimulation sessions. During the experimental task, participants were instructed to regulate their emotional responses while viewing each negative film clip by applying whichever of the three previously trained cognitive reappraisal strategies they considered most appropriate. Participants were free to choose the strategy on a trial-by-trial basis, irrespective of the stimulation condition. Only participants who demonstrated adequate understanding of the instructions and confidence in applying the strategies proceeded to the experimental phase. Additional details regarding the training procedures and instructional materials are provided in the Supplementary Materials (ER Training Guidelines – see article [38]).

### 2.5. Experimental sessions

During the experimental sessions, a set of measures and instruments was administered to assess the psychological impact of each video, the use of ER strategies, the overall psychological impact of the session, tACS tolerability, and to verify the integrity of the double-blind procedure.

#### 2.5.1. Pre- and post-task

The Positive and Negative Affect Schedule (PANAS [39],[40]) is a self-report instrument consisting of 20 adjectives designed to assess positive and negative emotional states. Participants rate each item using a 5-point Likert scale, ranging from 1 (very slightly or not at all) to 5 (extremely), based on their feelings. Both subscales showed adequate internal consistency, with Cronbach's α coefficients of 0.86 for positive affect and 0.89 for negative affect.

The Visual Analogue Scale (VAS) for tACS Side Effects is a self-report instrument that combines visual and numeric elements, using a 10-point Likert scale ranging from 0 (not at all) to 10 (extremely) to assess a range of psychophysiological symptoms frequently associated with non-invasive brain stimulation. This measure was used to monitor participant safety and assess the tolerability of the stimulation protocol.

The tACS Blinding Questionnaire was administered after each session to assess the efficacy of the double-blind procedure. Participants were asked to indicate which type of stimulation they believed they had received (active, sham, or unsure) and to rate their confidence in this judgment using a 5-point scale ranging from not at all confident to extremely confident. This tool was employed to verify the effectiveness of blinding and to minimize the influence of expectation-related biases on the study outcomes.

#### 2.5.2. During the task

Self-Assessment Manikin (SAM [41]) is a non-verbal, pictorial assessment instrument designed to directly assess subjective emotional responses, specifically valence and arousal levels. In this study, the 9-point version of the scale was used to evaluate participants' emotional reactions following each film.

List of Emotions: after each film, and following the SAM assessment, a list of 17 emotions was presented. Participants were instructed to select the emotion that best reflected their emotional experience while watching the film.

ER strategies: After selecting an emotion from the list, participants were asked whether they had used any ER strategy to manage the emotional response elicited by the film. The options included positive reappraisal, fictional reappraisal, decentralization, or simply observing the film.

### 2.6. Emotional induction task

The emotional induction paradigm was developed to elicit negative emotional reactions within a controlled laboratory environment using visual stimuli. This paradigm was crafted and executed using E-Prime software (version 3) and involved compiling brief film excerpts sourced from the Emotional Movie Database (EMDB [42],[43]).

During each experimental session, participants were presented with a total of 19 film clips: 11 with negative emotional content and 8 with neutral content. The task lasted approximately 25 minutes. Preceding the primary task, participants engaged in a concise training session involving three negative clips, during which they were instructed to use the three pre-trained CR strategies (one for each film), positive reappraisal, fictional reappraisal, or distancing, to familiarize themselves with the task framework and regulatory strategies.

Each trial followed a structured format: Participants first viewed a 5-second on-screen instruction, which either directed them to apply a CR strategy (for negative film clips) or to passively observe the film (for neutral clips). This was followed by the presentation of a 30-second film clip, and then a 35-second self-assessment phase. During the assessment, participants evaluated their emotional state using the SAM, rating valence and arousal. They were then asked to select the emotion that best described their emotional experience during the film from a predefined list. All clips were devoid of auditory accompaniment to obviate auditory influences, and the sequence of clips was randomized for each participant to decrease order effects. To prevent familiarity effects or emotional habituation, three distinct versions of the task (versions A, B, and C) were devised.

These versions followed the same overall structure and emotional content distribution but contained different film clips. Only the training phase incorporated identical stimuli among all versions. The configuration and temporal sequencing of task elements are delineated in Figure 1. For detailed descriptions of the EMDB film clips used, please refer to Table S1 in the Supplementary Material.

### 2.7. Transcranial alternating current stimulation

tACS was delivered using a DC-Stimulator Plus (NeuroConn GmbH, Germany). Stimulation was applied via two saline-soaked sponge electrodes (35 cm^2^, 7 × 5 cm) saturated in 0.9% NaCl solution to optimize conductivity and minimize skin resistance. Electrodes were positioned bilaterally over the dlPFC at F3 and F4, according to the international 10–20 EEG system (Figure 1C).

Participants completed three sessions: two active (0° in-phase and 180° anti-phase) and one sham. During the active sessions, a 2 mA peak-to-peak sinusoidal current at 10 Hz (alpha band) was applied for 20 min (≈20,000 cycles). Stimulation included a 15 s ramp-in and a 15 s ramp-out to reduce discomfort. This corresponded to a current density of 0.057 mA/cm^2^, which was well below the established safety thresholds.

In the sham condition, electrode placement was identical, but stimulation was limited to 60 s (15 s ramp-in, 30 s at 2 mA, 15 s ramp-out), after which the device deactivated automatically. This procedure was designed to mimic the initial sensations of active tACS and preserve participant blinding.

Throughout stimulation, electrode impedance was continuously monitored and maintained below 5 kΩ. All parameters were consistent with published safety recommendations for transcranial electrical stimulation [44].

To characterize the electric field distribution and verify safe targeting of the bilateral dlPFC montage, finite element method (FEM) simulations were conducted using SimNIBS 4.5.0 [45] on the standard “Ernie” head model. Rectangular sponge electrodes (70 × 50 mm) were positioned at F3 and F4 according to the international 10–20 EEG system. Simulations were performed using 1 mA opposing currents, corresponding to the 2mA peak-to-peak sinusoidal stimulation applied experimentally. Electric field magnitude (|E|) in gray matter showed a peak value of 0.196 V/m (99.9th percentile). Mean electric field strength within bilateral dlPFC regions was 0.089 V/m (left hemisphere) and 0.090 V/m (right hemisphere), indicating symmetrical field distribution across hemispheres. All estimated field magnitudes were well below established safety thresholds for cortical tissue (10 V/m; Radman et al., 2009), representing less than 2% of this limit. These simulations confirmed that the bilateral F3/F4 montage produces focal electric field engagement of prefrontal regions while remaining within conservative safety margins (Figure 2).

**Figure 2.**
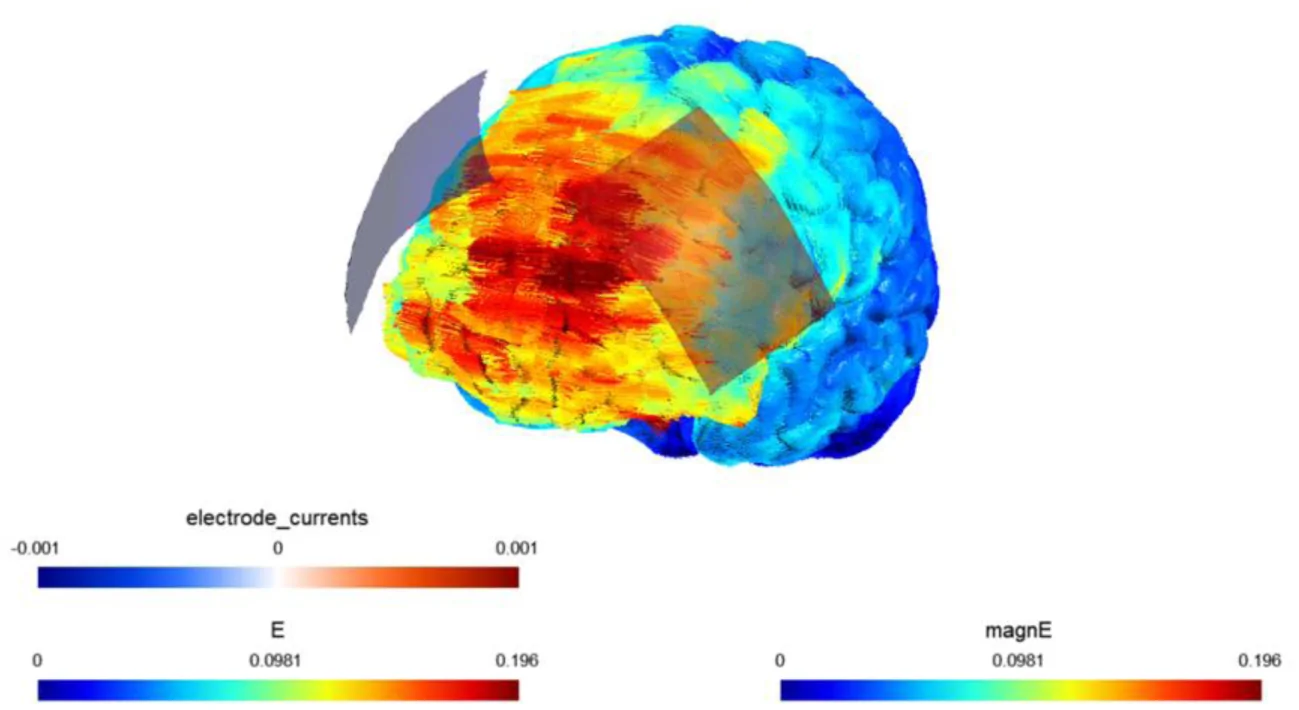
Computational Validation of Bilateral Dorsolateral Prefrontal Alpha-tACS: Electric Field Distribution. E, Electric field; magnE, Magnitude of Electric field (V/m).

### 2.8. Peripheral measures

Physiological signals were acquired and analyzed using LabChart software (version 8; Figure 1D). Signal processing was structured to reduce noise and eliminate artifacts, enabling the extraction of the most relevant components for subsequent analysis. To process heart rate data, artifacts caused by tACS signal or electrode instability were corrected using LabChart's arithmetic tool, with the Ch1/4 function applied to improve signal smoothness and reduce variability. For skin conductance, the analysis focused on separating the tonic level from the phasic responses. This was achieved by creating a new channel and applying the following function to clean the signal: Smoothsec (Differentiate (RClowpass (Ch5;0,205);3);1,3). The respiratory rate signal was used in its raw form, as it exhibited sufficient quality for reliable interpretation without additional processing (Figure 1B). Due to technical issues, data from three participants were excluded from the analysis of respiratory rates. After preprocessing, all signals were segmented by stimulus type (neutral vs. negative clips), and values were extracted in six 5-second windows (0–5, 5–10, 10–15, 15–20, 20–25, 25–30 s) to examine temporal dynamics.

### 2.9. Statistical analysis

Descriptive statistics were computed for all demographic and clinical variables. Continuous variables are reported as means and standard deviations, and categorical variables as frequencies and percentages.

Psychophysiological data were analyzed using linear mixed-effects models (LMMs), appropriate for repeated-measures designs with within-subject correlations and capable of accommodating unbalanced data and missing observations [46]. All models included random intercepts for participants to account for individual variability. Estimation was performed using restricted maximum likelihood (REML) with Satterthwaite approximations for degrees of freedom. Fixed-effect significance was assessed using Type III Wald F-tests, and Bonferroni-corrected post hoc comparisons were applied when required. Effect sizes were reported as Cohen's d for mean differences (0.20 small, 0.50 medium, 0.80 large) and partial eta squared (η^2^_p_) for omnibus tests (0.01 small, 0.06 medium, 0.14 large). Model adequacy was evaluated through marginal R^2^ (variance explained by fixed effects), conditional R^2^ (variance explained by fixed and random effects), and intraclass correlation coefficients (ICCs) reflecting between-person variability.

Models for subjective ratings (valence and arousal) included fixed effects for stimulation condition (sham, in-phase, anti-phase), film category (neutral, negative), and their interaction. Separate LMMs were computed for affective states (positive and negative affect) and for each of the 10 tDCS side-effects (anxiety, sadness, itch, headache, other pain, tingling, tiredness, agitation, sleepiness, metallic taste). These models included stimulation condition, time point (pre- vs. post-session), and their interaction as fixed effects, with participant entered as a random intercept.

For physiological measures (heart rate, skin conductance, and respiratory rate), dependent variables were computed as change scores, defined as the difference between responses to negative and neutral film clips (negative – neutral). This subtraction was used to control for individual differences in baseline autonomic activity and nonspecific reactivity, thereby isolating emotion-related physiological responses to negative stimuli. Fixed effects were stimulation condition (sham, in-phase, anti-phase) and time interval (six 5-second intervals: 0–5 s, 5–10 s, 10–15 s, 15–20 s, 20–25 s, 25–30 s) and two-way interactions. Orthogonal polynomial contrasts (linear and quadratic) were applied to the time-interval factor to characterize temporal trajectories [47]. Linear contrasts tested monotonic trends (e.g., habituation), while quadratic contrasts captured non-linear patterns (e.g., initial rise followed by decline). Data screening identified no extreme outliers (values > ±2.5 SD). Missing data were minimal: 0% for heart rate and skin conductance, and 2.3% for respiratory rate due to technical issues.

Blinding effectiveness was assessed using Bang's Blinding Index (BI [48]), computed separately for each stimulation condition. The BI ranged from −1 (systematic incorrect guessing) to 1 (systematic correct guessing), with 0 indicating random guessing (optimal blinding). Interpretation thresholds were: 0–0.2 good blinding, 0.2–0.4 moderate, 0.4–0.6 poor, and >0.6 very poor blinding [49]. “I don't know” responses were coded as incorrect in line with conservative blinding-assessment practices.

To quantify evidence for the absence of stimulation effects, Bayes factors (BF10) were computed for each primary analysis using the BayesFactor package [50], with a default Cauchy prior on standardised effect size (r = 0.707). BF10 values higher than 1 indicate evidence in favor of an effect, BF10 results lower than 1 indicate evidence in favor of no effect, and BF10 equal to 1 suggest that the data are ambiguous (Table S2).

## 3. Results

### 3.1. Sociodemographic and clinical data

Forty-two adults participated in this study (M_age = 19.83 years, SD = 1.55; 88.1% female). Most participants were Portuguese (97.6%), single (100%), and enrolled in higher education (81.0% full-time students and 19.0% working students). One participant reported a history of mental illness. Most reported no tobacco use (88.1%), and none reported alcohol consumption. Baseline psychometric measures indicated generally low levels of ER difficulties and psychological distress, with greater habitual use of cognitive reappraisal than expressive suppression. Detailed sociodemographic and psychometric characteristics are presented in Table 1. DERS-18 scores were interpreted dimensionally rather than as a clinical classification, given the ongoing debate regarding the factor structure and cutoff scores of the DERS [51].

**Table 1.** Sociodemographic and clinical characteristics.

	Group (N = 42)	
	M	SD / %
**Sociodemographic results**		
**Age**	19.83	1.55
**Sex**		
Female	37	88.10
Male	5	11.90
**Nationality**		
Portuguese	41	97.60
Brazilian	1	2.40
**Marital status**		
Single	42	100
**Education**		
High school	42	100
**Occupation**		
Student	34	81.00
Worker student	8	19.00
**Clinical results**		
**Mental illness**		
Yes	1	2.40
No	41	97.60
**Alcohol use**		
No	42	100
**Tobacco use**		
Yes	5	11.90
No	37	88.10
**Clinical results**		
**ER difficulties**	38.88	10.55
Awareness	5.74	2.26
Clarity	5.88	1.74
Goals	10.17	2.90
Impulse	5.29	2.82
Nonacceptance	5.55	2.23
Strategies	6.26	2.92
**ER strategies**		
Cognitive reappraisal	28.31	7.88
Expressive suppression	12.71	5.54
**Cognitive ER**		
Acceptance	12.52	3.32
Positive refocusing	11.50	4.55
Refocus on planning	13.52	3.94
Positive reappraisal	13.40	4.22
Putting into perspective	12.10	3.69
Self-blame	9.33	3.24
Rumination	13.26	3.34
Catastrophizing	7.57	2.89
Blaming others	6.93	2.56
**Mental illness**		
Anxiety	4.86	4.78
Depression	4.26	4.44
Stress	7.86	4.80
**Social desirability**	18.29	4.68

### 3.2. Self-report measures: Valence and arousal

Valence and arousal responses to the film clips were analyzed using linear mixed-effects models (LMMs) with stimulation condition (sham, in-phase, anti-phase) and film category (neutral, negative) as fixed effects and participant as a random intercept (Figure 3). Descriptive statistics, model fit indices, and complete model outputs are presented in Supplementary Tables S3–S5.

For valence, a significant main effect of film category was observed, F(1, 205) = 846.80, p < 0.001, η²_p_ = 0.805, indicating that neutral films were rated more positively than negative films (β = −2.06, SE = 0.12, 95% CI [−2.29, −1.82], t(205) = −17.05, p < 0.001, d = −2.55). Neither the main effect of stimulation condition, F(2, 205) = 0.65, p = 0.522, η²_p_ = 0.006, nor the interaction between film category and stimulation condition, F(2, 205) = 0.29, p = 0.750, η²_p_ = 0.003, was statistically significant.

Similarly, arousal ratings showed a significant main effect of film category, F(1, 205) = 431.45, p < 0.001, η²_p_ = 0.678, with negative films eliciting higher arousal than neutral films (β = 2.78, SE = 0.23, 95% CI [2.33, 3.24], t(205) = 11.96, p < 0.001, d = 1.74). Neither the main effect of stimulation condition, F(2, 205) = 0.08, p = 0.922, η²_p_ = 0.001, nor the interaction between film category and stimulation condition, F(2, 205) = 0.11, p = 0.896, η^2^_p_ = 0.001, was statistically significant (see Table S5). Additional self-reported emotional outcomes are reported in Description S2 (Table S6).

**Figure 3.**
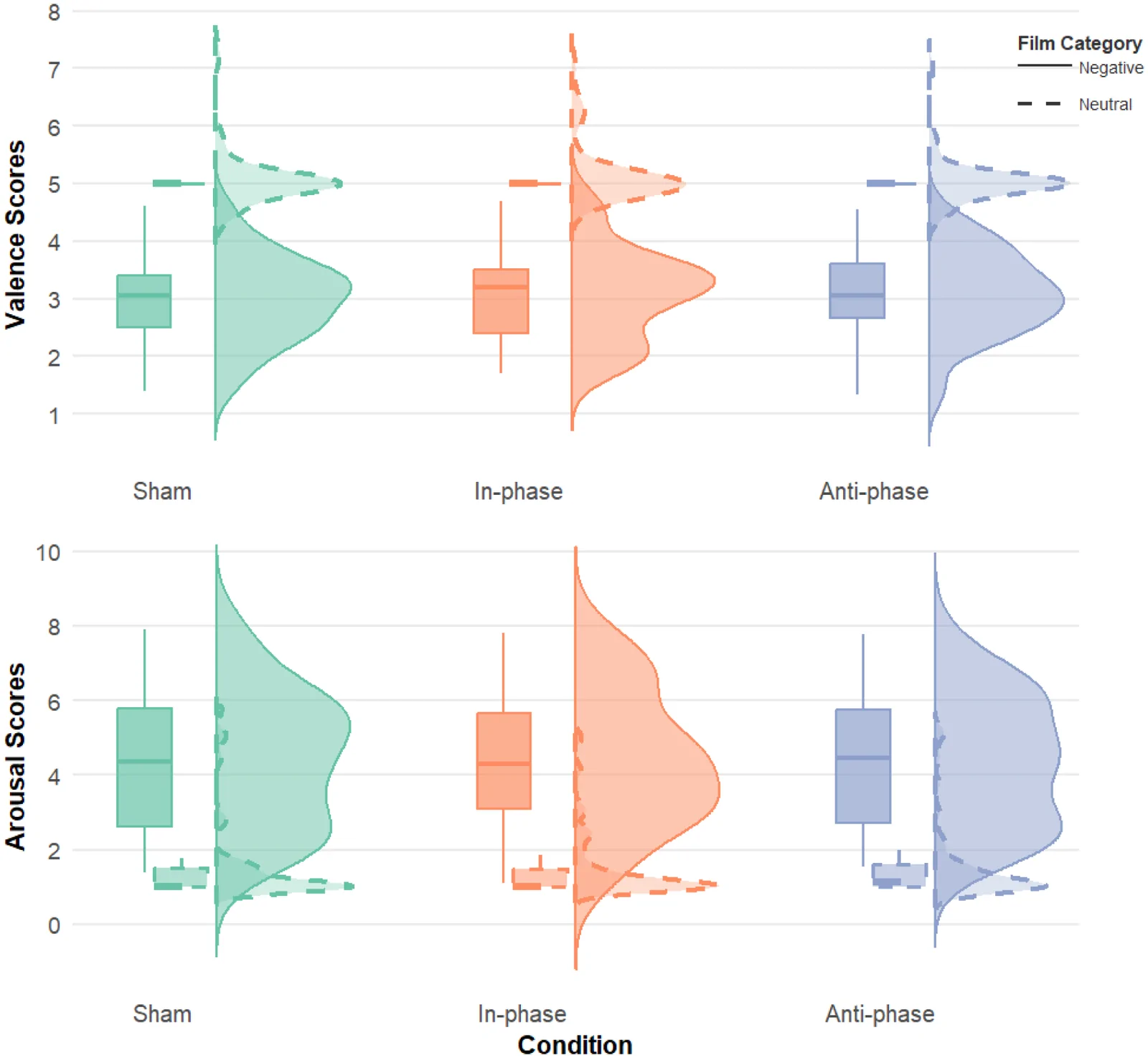
Emotional responses – valence and arousal – across conditions: quartiles and distributions.

### 3.3. Physiological measures: Differences in physiological activation across time

The effects of time and stimulation conditions on emotional reactivity in heart rate (HR), skin conductance (SC), and respiratory rate (RR) were explored using LMMs (see Table S7 for descriptive statistics). To isolate emotional reactivity, difference scores were calculated by subtracting neutral film responses from negative film responses at each time interval for each participant and condition (negative – neutral) (see Table S8). The LMMs included fixed factors for stimulation condition (sham, in-phase, anti-phase) and time interval (six 5-second intervals: 0–5 s, 5–10 s, 10–15 s, 15–20 s, 20–25 s, 25–30 s), as well as their two-way interaction (stimulation condition × time interval).

#### 3.3.1. Differences between conditions – Heart Rate

The complete HR difference score model explained 40.7% of variance in HR reactivity (conditional R^2^ = 0.407), with fixed effects alone accounting for 1.2% of variance (marginal R^2^ = 0.012). Variance components indicated considerable between-participants variance (τ^2^_00_ = 0.09), participant-by-condition variance (τ^2^_01_ = 4.46), and residual variance (σ^2^ = 6.83). An ICC of 0.400 indicated that 40.0% of total variance was attributable to individual differences (see Table S9).

Type III tests revealed no significant major effects or interactions. The main effect of stimulation condition, F(2, 82) = 0.05, p = 0.955, η^2^_p_ = 0.001, time interval, F(5, 615) = 1.01, p = 0.411, η^2^_p_ = 0.008 and condition × time interval interaction were not significant, F(10, 615) = 0.95, p = 0.488, η^2^_p_ = 0.020 (see Table S10).

Polynomial contrast analyses examining temporal trends in HR reactivity revealed no significant linear or quadratic patterns within any stimulation condition. In the sham condition, there was a marginal linear decrease (β = −0.24, t = −1.76, p = 0.080) and no quadratic trend (β = 0.03, t = 0.30, p = 0.766). The in-phase condition showed no significant linear trend (β = 0.08, t = 0.64, p = 0.523) or quadratic trend (β = −0.08, t = −0.91, p = 0.365). Similarly, the anti-phase condition showed no significant linear (β = −0.11, t = −1.07, p = 0.288) or quadratic trends (β = −0.06, t = −0.78, p = 0.434). These findings indicated that HR reactivity to emotional stimuli remained relatively stable across the 30-second viewing window, regardless of stimulation condition (Figure 4).

**Figure 4.**
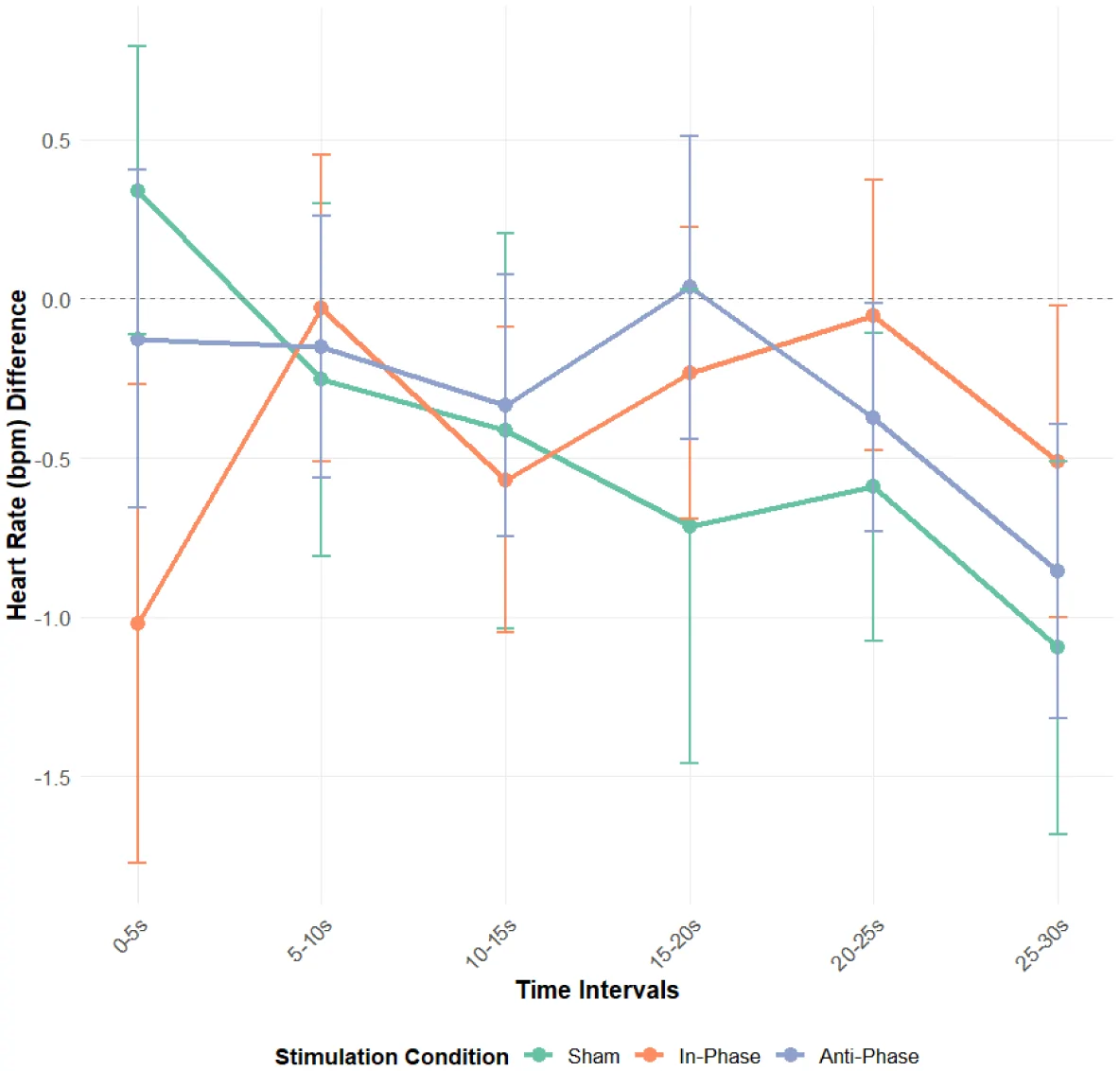
Mean heart rate difference scores (Negative - Neutral) across time intervals under three stimulation conditions (sham, in-phase, anti-phase). Error bars represent standard errors.

#### 3.3.2. Differences between conditions - Skin Conductance

The complete SC difference score model explained 4.2% of variance (conditional R^2^ = 0.042), with fixed effects accounting for 4.1% (marginal R^2^ = 0.041). Note that the model showed a singular fit with near-zero between-participant variance (τ^2^_00_= 0.00), between-participant variance (τ^2^_00_ = 0.00), and residual variance (σ^2^ = 0.01). An ICC of 0.001 indicated minimal individual variability in SC reactivity difference scores (see Table S9).

There was a significant main effect of time interval, F(5, 697) = 3.01, p = 0.011, η^2^_p_ = 0.021, with SC reactivity modifying across the film (β = −0.04, SE = 0.02, 95% CI [−0.07, −0.00], t(697) = −2.16, p = 0.031, d = −0.16). The main effect of stimulation condition was not significant, F(2, 697) = 0.68, p = 0.506, η^2^_p_ = 0.002. The stimulation condition × time interval interaction was also not significant, F(10, 697) = 1.58, p = 0.107, η^2^_p_ = 0.022 (see Table S11).

Polynomial contrast analyses revealed significant linear decreases in SC reactivity over time in sham (β = −0.01, t = −2.30, p = 0.022) and in-phase conditions (β = −0.01, t = −2.51, p = 0.013), but not in the anti-phase condition (β = −0.00, t = −1.07, p = 0.284). The quadratic trend was not significant in all conditions: sham (β = 0.00, t =1.28, p = 0.202), in-phase (β = 0.00, t = 0.77, p = 0.440), and anti-phase (β = −0.00, t = −0.86, p = 0.393).

Post-hoc analyses with Bonferroni correction examined pairwise differences across time intervals. SC reactivity during the first interval (0–5 s) was significantly higher than during the last interval (25–30 s), with mean a difference = 0.053 µS, SE = 0.014, p = 0.004, and d = 0.47 (Figure 5).

**Figure 5.**
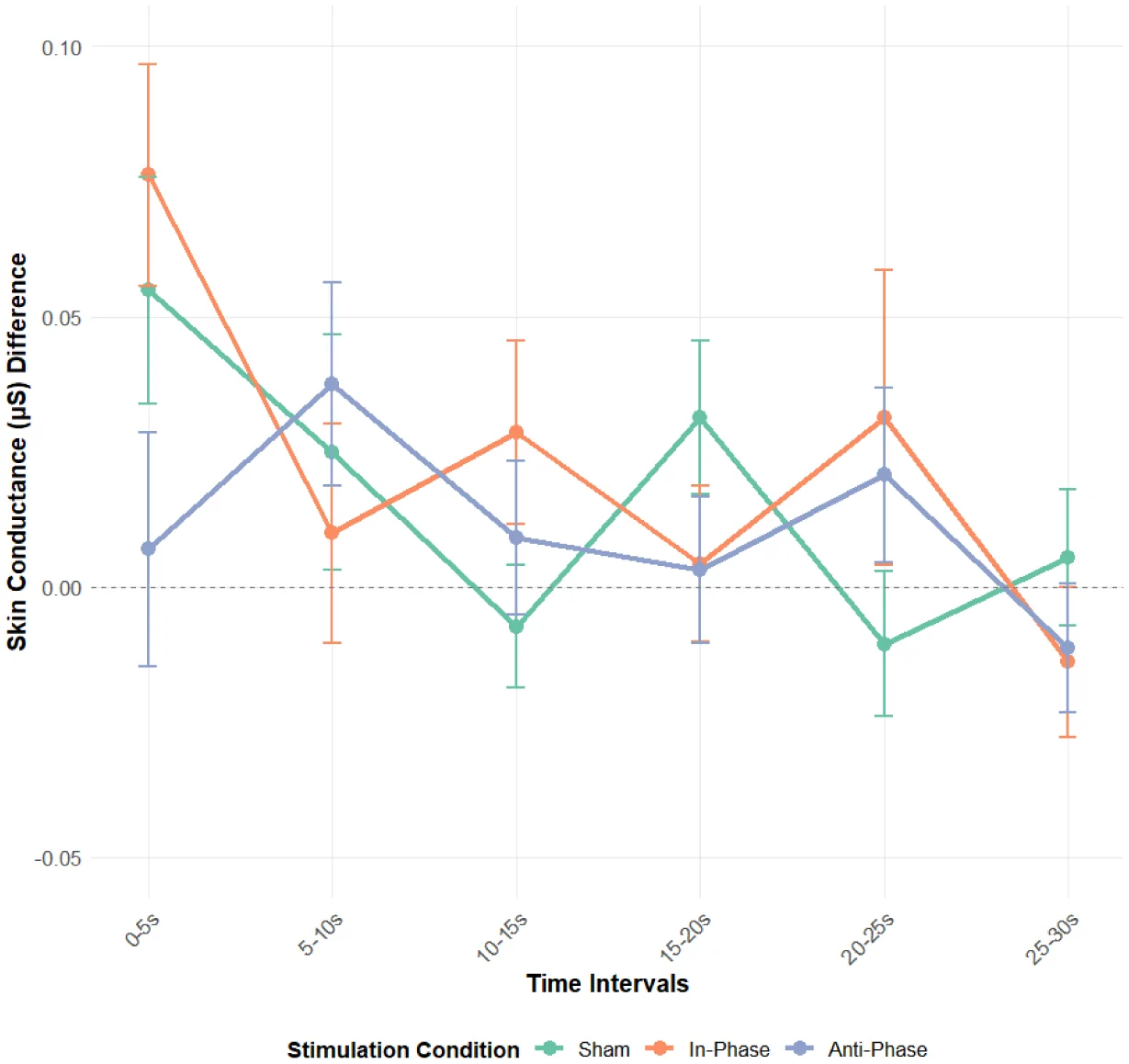
Mean skin conductance difference scores (Negative - Neutral) across time intervals under three stimulation conditions (sham, in-phase, anti-phase). Error bars represent standard errors.

#### 3.3.3. Differences between conditions - Respiratory Rate

The complete RR difference score model explained 49.2% of variance (conditional R^2^ = 0.492), with fixed effects accounting for 1.8% (marginal R^2^ = 0.018). Variance components were σ^2^ participant = 1.18, σ^2^ participant × condition = 0.79, and σ^2^ residual = 2.10 (SD = 1.45). An ICC of 0.483 indicated substantial individual variability, with approximately 48% variance attributable to individual differences (see Table S9).

There was a highly significant main effect of time interval, F(5, 595.54) = 3.28, p = 0.006, η^2^_p_ = 0.027, reflecting temporal dynamics in RR reactivity, β = −0.51, SE = 0.22, 95% CI [−0.95, −0.07], t(596) = −2.27, p = 0.024, d = 0.19. The main effect of stimulation condition was not significant, F(2, 79.96) = 0.05, p = 0.949, η^2^_p_ = 0.001. The stimulation condition × time interval interaction was also not significant, F(10, 595.54) = 0.85, p = 0.584, η^2^_p_ = 0.014 (see Table S12).

Polynomial contrast analyses revealed no significant linear or quadratic patterns within any stimulation condition: linear trends - sham (β = −0.09, t = −1.20, p = 0.231), in-phase (β = 0.07, t = 1.03, p = 0.306), or anti-phase (β = −0.01, t = −0.16, p = 0.875); quadratic trends − sham (β = −0.08, t = −1.55, p = 0.121), in-phase (β = −0.06, t = −1.32, p = 0.187), or anti-phase (β = −0.03, t = −0.60, p = 0.547).

Post-hoc analyses with Bonferroni correction revealed significant temporal changes in RR reactivity. RR reactivity at the first interval (0–5 s) was significantly lower than at the second interval (5–10 s), mean difference = −0.64 breaths/min, SE = 0.19, p = 0.009, d = −0.44, and significantly lower than at the third interval (10–15 s), mean difference = −0.59 breaths/min, SE = 0.19, p = 0.024, d = −0.41, indicating an initial rise in respiratory reactivity during the first 10–15 seconds of emotional film viewing (Figure 6).

**Figure 6.**
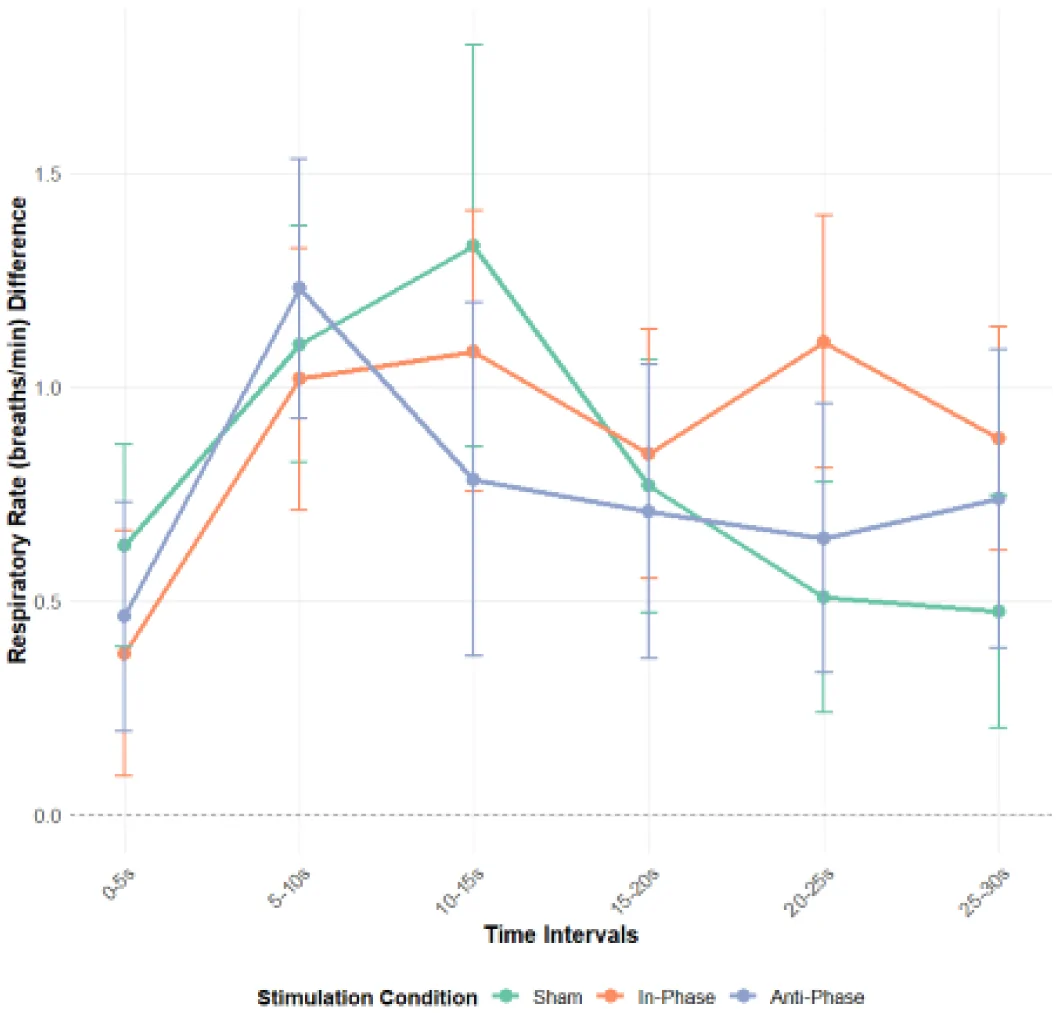
Mean respiratory rate difference scores (Negative - Neutral) across time intervals under three stimulation conditions (sham, in-phase, anti-phase). Error bars represent standard errors.

### 3.4. Changes in self-report measures (Pre–post)

#### 3.4.1. Positive and negative affect

LMMs were conducted to explore the effects of stimulation condition (sham, in-phase, anti-phase) and time point (pre-session, post-session) on self-reported affect, with separate analyses for positive and negative affect scores (see Table S13 for descriptive statistics). Both models included participant as a random intercept to account for repeated measures.

Model fit indices indicated that fixed effects explained minimal variance in positive affect scores (marginal R^2^ = 0.029), but substantial variance was explained when including random effects (conditional R^2^ = 0.708). Variance components revealed substantial between-participants variance (τ^2^_00_ = 42.05, SD = 6.48) and residual variance (σ^2^ = 18.11, SD = 4.26). For negative affect, fixed effects explained minimal variance (marginal R^2^ = 0.017), with modest total variance explained (conditional R^2^ = 0.210). Between-participants variance was low (τ^2^_00_ = 1.91, SD = 1.38), with larger residual variance (σ^2^ = 7.84, SD = 2.80). An ICC of 0.699 demonstrated substantial between-person variability for positive affect and low between-person variability for negative affect (ICC = 0.196) (Figure 7; see Table S14).

The LMM for positive affect revealed a significant main effect of time, F(1, 205) = 23.58, p < 0.001, η^2^_p_ = 0.100, indicating that positive affect scores decreased from pre-session (M = 28.53, SD = 7.45) to post-session (M = 25.97, SD = 7.99), β = −2.60, SE = 0.54, t(205) = −4.86, p < 0.001, d = 0.34. The main effect of stimulation condition was not significant, F(2, 205) = 0.58, p = 0.561, η^2^_p_ = 0.005, nor was the condition × time interaction, F(2, 205) = 0.28, p = 0.759, η²_p_ = 0.003 (Table S15).

The LMM for negative affect revealed no significant main effect of time, F(1, 205) = 1.48, p = 0.226, η^2^_p_ = 0.007, indicating that negative affect scores remained relatively stable from pre-session (M = 12.67, SD = 3.19) to post-session (M = 12.24, SD = 3.13), β = −0.43, SE = 0.35, t(205) = −1.21, p = 0.226, d = 0.14. The main effect of stimulation condition was not significant, F(2, 205) = 1.34, p = 0.264, η^2^_p_ = 0.010, nor was the condition × time interaction, F(2, 205) = 0.57, p = 0.568, η^2^_p_ = 0.006 (Table S16).

**Figure 7.**
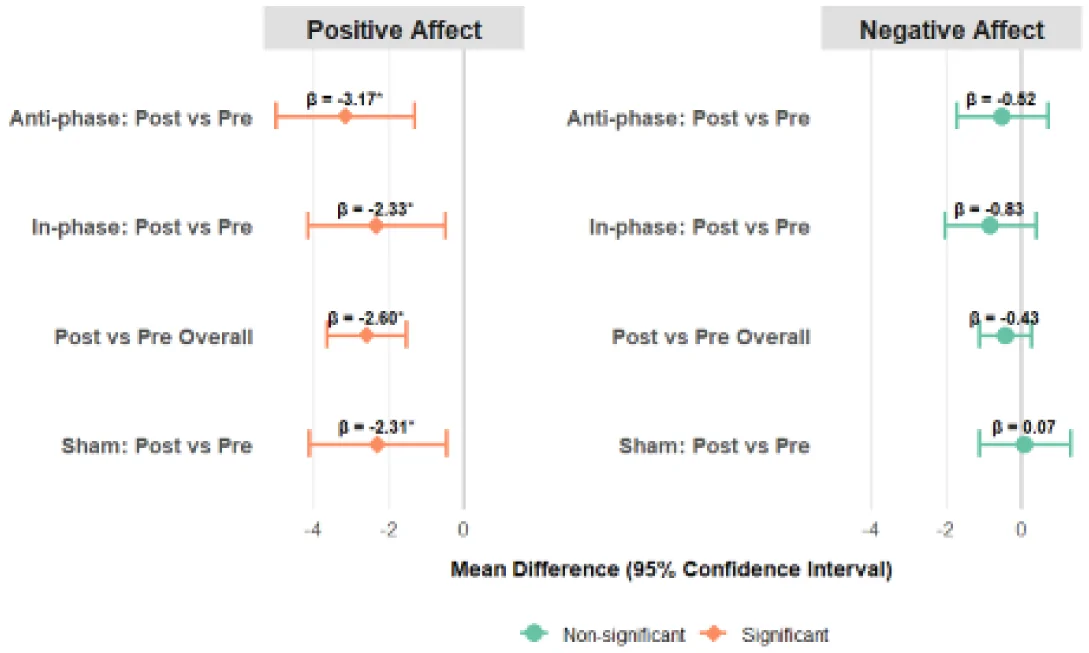
Mean Differences in Positive and Negative Affect: Time Effects and Condition Comparisons. Error bars represent 95% confidence intervals. The four rows show time effects (post- vs. pre-session): overall and separately for each stimulation condition. Asterisks indicate statistically significant differences (p < 0.05).

### 3.5. tACS side effects and blinding

#### 3.5.1. tACS side effects

Linear mixed-effects models were used to assess whether stimulation condition (sham, in-phase, anti-phase) and time (pre- vs. post-session) influenced self-reported side effects, with separate models fitted for each outcome and participant included as a random intercept.

Of the ten side effects evaluated, four showed significant changes over time, anxiety, itch, headache, and tingling, but none differed across stimulation conditions, and no time × condition interactions emerged. Fixed effects explained little variance, with most variability occurring within individuals rather than between participants (see Tables S17–19). Anxiety decreased from pre- to post-session, whereas itch, headache, and tingling showed small increases.

All other side effects (sadness, other pain, tiredness, agitation, sleepiness, metallic taste) showed no significant effects. Overall, side effects were low in intensity, consistent across conditions, and followed typical pre–post fluctuations expected in tACS protocols.

#### 3.5.2. Blinding effectiveness

Bang's blinding index results showed different blinding effectiveness across the three stimulation conditions. The sham condition demonstrated poor blinding effectiveness (BI = 0.571), indicating that participants showed systematic response bias rather than random guessing. In contrast, both experimental conditions showed moderate blinding: in-phase stimulation (BI = 0.381) and anti-phase stimulation (BI = 0.286). For the sham condition, 15 participants (35.7%) correctly identified the type of stimulation. The in-phase condition showed better discrimination, with 25 participants (59.5%) correctly identifying experimental stimulation. Similarly, for the anti-phase condition, 24 participants (57.1%) correctly identified experimental stimulation (Figure S1).

## 4. Discussion

In this study, we examined whether in-phase and anti-phase alpha-band tACS applied over the dlPFC could enhance cognitive reappraisal and modulate autonomic responses during emotional film viewing. Contrary to our hypotheses, neither active stimulation condition altered subjective emotional ratings, autonomic indices (heart rate, skin conductance, or respiratory rate), nor pre- to post-session affective changes compared with sham stimulation. Instead, emotional film category robustly influenced subjective ratings, and physiological responses showed the expected temporal adaptation across stimulus presentation, indicating that the emotional paradigm functioned as intended while the stimulation protocol produced no detectable modulatory effects. These findings indicated that the stimulation protocol used in this study did not produce measurable enhancement of ER. This interpretation was further strengthened by the Bayesian analyses, which consistently provided evidence favoring the null hypothesis over stimulation-related effects. The convergence of frequentist and Bayesian approaches strengthens confidence that no measurable behavioral or autonomic effects of the stimulation protocol were observed under the experimental conditions tested. To our knowledge, we are the first to examine phase-specific alpha-tACS during cognitive reappraisal using ecologically valid film stimuli, providing important evidence regarding the boundary conditions under which frontal alpha-tACS may influence ER.

Regarding self-report measures, the expected effects of film category emerged: Negative clips were rated as less pleasant and more arousing than neutral clips. However, stimulation condition did not influence valence or arousal ratings, nor did it interact with film category. These findings converge with literature showing that CR does not always alter the emotional perception of negative scenes [52] and align with evidence that PFC transcranial electrical stimulation does not consistently modify emotional bias or subjective affect [53].

Physiological results mirrored the subjective observations, demonstrating minimal influence of the stimulation condition. Across the three conditions (sham, in-phase, anti-phase), HR, SC, and RR remained largely unaffected, either as main effects or in interaction with time. Instead, the physiological response pattern was primarily shaped by stimulus dynamics, with modest time-dependent changes observed in SC and RR. These findings suggest that the physiological response to emotional stimuli is primarily influenced by temporal processes, and not by the stimulation parameters tested in this study. Collectively, these autonomic findings indicate that emotional responding was primarily governed by the intrinsic temporal dynamics of each physiological system rather than by phase-specific neuromodulation. Heart rate remained stable throughout the task, whereas skin conductance exhibited the expected habituation profile and respiratory rate showed a transient early increase, patterns that are consistent with well-established autonomic responses to sustained emotional stimulation.

Heart rate showed no modulation by stimulation condition or time, indicating largely stable cardiac response throughout film viewing. This absence of change aligns with findings from May et al., who also reported no effects of alpha- or gamma-tACS (versus sham) on autonomic indices, including HR and SC, even after multiple sessions. The relatively high intraclass correlation coefficient (ICC ≈ 0.40) observed here further indicates that interindividual differences accounted for a substantial proportion of variance in HR, consistent with well-established evidence that cardiac activity is strongly shaped by person-specific psychophysiological factors [54]. Such heterogeneity may markedly reduce sensitivity to subtle neuromodulatory effects unless sample sizes are large or participants are stratified on relevant traits.

In contrast, SC demonstrated a temporal pattern: A small but systematic decline across the 30 s clips. Post-hoc comparisons confirmed that electrodermal reactivity was highest during the first 5 seconds and progressively decreased, consistent with the characteristic rapid peak and subsequent habituation of phasic sympathetic responses to emotional stimuli [55]. SC is highly sensitive to stimulus novelty and salience and declines even when emotional intensity remains constant. The very low ICC observed for SC suggests minimal between-person variability in these difference scores, indicating that electrodermal responses were strikingly homogeneous across participants. Although this reduced between-participant variability would generally increase sensitivity to detect systematic stimulation effects, no such effects were observed. Instead, the consistent pattern of electrodermal habituation across participants suggests that skin conductance responses were primarily driven by the intrinsic temporal dynamics of emotional processing rather than by the stimulation protocol.

Respiratory rate also showed modest temporal modulation, with an initial increase during the early intervals followed by stabilization. This initial trajectory is consistent with typical emotion-related respiratory activation, where early orienting responses are followed by increased respiratory drive during emotional engagement [56]. Similar to HR, the relatively high ICC (≈0.48) indicates strong interindividual variability in respiratory reactivity, likely reflecting differences in baseline respiratory patterns, anxiety sensitivity, or voluntary respiratory control. This heterogeneity, combined with fixed effects explaining less than 2% of total variance, likely reduced the power to detect subtle stimulation-related effects.

Although this study did not reveal measurable effects of phase-specific alpha-tACS during cognitive reappraisal, several factors may account for these null findings. One possibility is that phase-dependent modulation is more likely to emerge under conditions requiring strong engagement of executive-control networks. For instance, Seo et al. [57] demonstrated behavioral effects of phase-specific stimulation during a Stroop task involving high levels of conflict processing. However, because we did not directly quantify cognitive load or executive-control demands, this comparison should be interpreted cautiously. Rather than providing a direct explanation for our findings, these observations suggest that the behavioral effects of phase-specific tACS may depend on the interaction between stimulation parameters and task characteristics. Additional factors, including variability in endogenous oscillatory dynamics, individual alpha frequency, and the absence of direct verification of neural entrainment, may also have reduced the likelihood of observing measurable behavioral or autonomic effects.

In addition, our findings stand in contrast to our previous tDCS study using the same film-based paradigm [38], in which anodal dlPFC stimulation produced significant reductions in heart rate during reappraisal and even in the absence of explicit CR instructions. This divergence may reflect differences in the neurophysiological mechanisms engaged by these neuromodulation techniques, although direct comparisons should be interpreted cautiously because the studies differed in stimulation protocols and experimental conditions. One possible explanation is that tDCS and open-loop tACS influence cortical activity through partially distinct neurophysiological mechanisms. Whereas tDCS is generally thought to induce relatively sustained changes in cortical excitability, the behavioral effects of open-loop tACS are considered to depend more strongly on successful oscillatory entrainment and alignment with endogenous brain rhythms. Consequently, the absence of measurable autonomic effects in this study suggests that researchers should explore more sensitive stimulation approaches, including multi-session protocols, individualized alpha-frequency tuning, and closed-loop stimulation synchronized to ongoing neural activity. Taken together, the available evidence suggests that tDCS and open-loop tACS may engage partially distinct neurophysiological processes during ER. Whereas tDCS is generally thought to facilitate relatively sustained changes in cortical excitability, the behavioral effects of open-loop tACS may depend more strongly on successful oscillatory entrainment and precise phase alignment.

Another possible explanation for the absence of tACS effects relates to the interaction between the stimulation protocol and endogenous brain oscillations. The bilateral 10-Hz stimulation protocol may have been insufficiently matched to participants' endogenous oscillatory dynamics. Growing evidence suggests that the behavioral effects of tACS depend critically on the correspondence between stimulation frequency and individual intrinsic rhythms, as well as on the ongoing brain state during stimulation. Variability in individual alpha frequency may therefore have attenuated neural entrainment, reducing the likelihood of detecting measurable behavioral or autonomic effects.

In the pre- and post-session assessments, positive affect decreased significantly across all stimulation conditions, indicating a general reduction in positive mood following exposure to the emotional task. Although participants receiving active stimulation showed numerically lower negative affect scores than those in the sham condition, these differences were small and did not reach statistical significance. Overall, these findings are consistent with evidence indicating that the effects of prefrontal transcranial electrical stimulation on affective processing are often subtle and context dependent [24],[58],[59]. The apparent discrepancy between the trial-by-trial emotional ratings and the pre–post PANAS results likely reflects differences in the temporal characteristics of the two measures. The SAM captured immediate emotional responses to individual film clips, whereas the PANAS assessed broader affective states across the experimental session. As a result, any subtle or short-lived effects of alpha-tACS occurring during individual reappraisal trials may have been diluted when affect was evaluated as a global state after completion of the session.

Overall, we did not detect measurable effects of phase-specific alpha-tACS on subjective or autonomic indices of ER under the experimental conditions employed. However, these null findings should be interpreted cautiously. Because cognitive reappraisal implementation was not monitored on a trial-by-trial basis, concurrent EEG was not available to verify neural entrainment, and sham blinding was imperfect, we cannot determine whether the absence of behavioral effects reflects unsuccessful target engagement, variability in strategy implementation, limitations of the stimulation protocol, or a genuine absence of stimulation effects. Consequently, our findings should not be considered conclusive regarding the causal role of phase-specific alpha oscillations in cognitive reappraisal. Future studies incorporating online strategy monitoring, concurrent electrophysiological recordings, and improved sham procedures will be necessary to disentangle these alternative explanations.

Finally, this investigation has several limitations that should be considered when interpreting our results. The sample was predominantly female (88%), preventing meaningful evaluation of potential sex-related differences in emotional processing or responsiveness to alpha-tACS. Although this homogeneity reduced variability in this initial study, future investigations with more balanced samples will be necessary to determine whether sex moderates behavioral or autonomic responses to phase-specific stimulation. However, this narrow demographic profile limits representativeness and, consequently, reduces the generalizability of the findings to broader populations. Moreover, although participants were trained in three validated cognitive reappraisal strategies (positive reappraisal, fictional reappraisal, and distancing), strategy use was not monitored on a trial-by-trial basis during the experimental task. Consequently, differences in strategy selection or implementation may have introduced uncontrolled variability, potentially obscuring subtle behavioral or physiological effects of stimulation. Furthermore, the reliance on self-report measures may have introduced social desirability bias, potentially influencing how participants reported their emotional experiences [60]. In addition, blinding was less effective in the sham condition than in the active stimulation conditions, as indicated by the Bang Blinding Index. Although no significant differences between stimulation conditions were observed, incomplete blinding may have introduced expectancy-related effects that should be considered when interpreting our findings. Future studies should formally examine whether participants' beliefs about the stimulation condition influence behavioral or physiological outcomes. The use of silent film clips, although ecologically valid, may also have reduced emotional intensity compared with fully immersive audiovisual stimuli. Furthermore, the we did not include concurrent EEG or other neurophysiological measures capable of verifying target engagement, confirming successful neural entrainment, or assessing whether frontal alpha asymmetry (FAA) and interhemispheric phase synchrony were modulated as intended. Although the stimulation protocol was designed to synchronize bilateral alpha oscillations and manipulate phase relationships between prefrontal regions, we cannot determine whether these neural effects were successfully induced in our participants. Consequently, the absence of behavioral and autonomic effects should not be interpreted as evidence that phase-specific alpha-tACS is ineffective for ER. Rather, unsuccessful or insufficient neural entrainment remains a plausible explanation for our null findings. Therefore, we cannot distinguish a genuine absence of behavioral effects despite successful modulation of neural activity and a failure of the stimulation protocol to effectively entrain the targeted oscillatory networks. Future studies integrating concurrent EEG with phase-specific tACS will be essential to directly verify neural target engagement, quantify changes in frontal alpha asymmetry and interhemispheric synchrony, and establish more precise relationships between neural and behavioral responses. Finally, the modest sample size may have reduced statistical power, particularly for autonomic measures that exhibited substantial interindividual variability.

Future studies should aim to recruit more diverse participant groups, monitor and quantify participants' use of specific cognitive reappraisal strategies during the task, and employ more immersive emotional stimuli to increase affective engagement. Integrating concurrent EEG would enable direct verification of stimulation-induced neural entrainment, changes in frontal alpha asymmetry, and interhemispheric phase synchronization, thereby enabling closer links between neural and behavioral effects to be established. Larger sample sizes will also be essential for detecting subtle autonomic effects and for examining potential moderators, such as trait anxiety, baseline affective reactivity, and individual alpha frequency.

From a mechanistic perspective, future research should extend beyond alpha oscillations and examine the effects of theta-frequency (4–8 Hz) tACS over prefrontal regions. Frontal midline theta has been consistently implicated in cognitive control, conflict monitoring, and the dynamic coordination of distributed executive-control networks, particularly involving the dlPFC and dorsal anterior cingulate cortex. Theta-band synchronization is thought to facilitate long-range communication between prefrontal and limbic regions during effortful ER, whereas alpha oscillations are more closely associated with inhibitory gating and modulation of cortical excitability. Researchers should also investigate individualized stimulation protocols, including stimulation based on individual alpha frequency and closed-loop approaches synchronized to ongoing neural activity, to optimize oscillatory entrainment. Together, these methodological and mechanistic refinements may help clarify the conditions under which phase-specific tACS can meaningfully influence ER.

## Use of AI tools declaration

During the preparation of this work, the authors used ChatGPT-5.1 to assist with language refinement and organization of text. After using this tool, the authors thoroughly reviewed, edited, and verified all generated content to ensure accuracy, originality, and full alignment with the scientific aims and interpretations of the study. The authors take full responsibility for the integrity and content of the final manuscript.


