# Supplementary material for: Phase-specific alpha tACS over the dlPFC does not enhance cognitive reappraisal or autonomic responses: A sham-controlled crossover study
Source: AIMS Neurosci. 2026 Aug 26;13(3):483–509. doi: 10.3934/Neuroscience.2026022 (PMC13658056; doi:10.3934/Neuroscience.2026022)
Supplement: Supplementary file 1 [file neurosci-13-03-022-s001.pdf]

AIMS Neuroscience

AIMS Neuroscience, 13(3): 483–509.
DOI: 10.3934/Neuroscience.2026022
Received: 12 March 2026
Revised: 06 July 2026
Accepted: 15 July 2026
Published: 26 August 2026

https://www.aimspress.com/journal/neuroscience

Research article

Phase-specific alpha tACS over the dlPFC does not enhance cognitive reappraisal or autonomic responses: A sham-controlled crossover study

Catarina Gomes Coelho[1,2], Jorge Leite[3], Gabriela Gonzaga[1], Paulo Machado[2] and Sandra Carvalho[1,*]

[1] Psychology Research Centre (CIPsi), Department of Basic Psychology, School of Psychology, University of Minho, Braga, Portugal
[2] Psychology Research Centre (CIPsi), Department of Applied Psychology, School of Psychology, University of Minho, Braga, Portugal
[3] CINTESIS@RISE, CINTESIS.UPT, Portucalense University, Porto, Portugal

* **Correspondence:** Email: sandrarc@psi.uminho.pt.

Supplementary

Description S1. Instruments

Handedness was assessed using the Edinburgh Handedness Inventory (EHI 31) a 10-item self-report inventory originally developed by Oldfield 32 that evaluates hand preference across everyday activities. Scores range from −100 (indicating strong left-handedness) to +100 (indicating strong right-handedness). The Portuguese version showed robust internal consistency, with a Cronbach's α of 0.88.

The Difficulties in Emotion Regulation Scale - Short Form (DERS-18 33) is a self-report instrument that uses a 5-point Likert scale ranging from 1 (almost never) to 5 (almost always) to assess ER difficulties during negative emotion experiences. The validation showed high internal consistency values, with a Cronbach's α of 0.93.

The Emotion Regulation Questionnaire (ERQ-10 3) is a self-report instrument that uses a 7-point Likert scale, ranging from 1 (strongly disagree) to 7 (strongly agree), to assess individuals' tendency to use cognitive reappraisal and expressive suppression strategies. Both subscales showed adequate

internal consistency, with Cronbach's α coefficients of 0.82 for cognitive reappraisal and 0.72 for expressive suppression.

The Cognitive Emotion Regulation Questionnaire (CERQ-36 34,35) is a self-report instrument that uses a 5-point Likert scale ranging from 1 (almost never) to 5 (almost always) to assess individual differences in the use of cognitive ER strategies following negative or stressful life events. The validation results showed high internal consistency values, with Cronbach's α ranging from 0.75 to 0.91 across the subscales.

The Depression Anxiety Stress Scale (DASS-21 36) is a self-report instrument that uses a 4-point Likert scale ranging between 0 (did not apply to me at all) and 3 (applied to me very much or most of the time) to measure the presence of depression, anxiety, and stress symptoms. All subscales and the total scale presented adequate internal consistency, with Cronbach's α scores ranging between 0.82 and 0.93.

The Marlowe-Crowne Social Desirability Scale (MCSDS-33 37) is a self-report instrument consisting of true-or-false items designed to evaluate the tendency to respond in a socially desirable manner. The validation study demonstrated satisfactory internal consistency, with Cronbach's of 0.78.

Table S1. Descriptions of the film clips used from the EMDB.

Categories	Version A		Valence M (SD)	Arousal M (SD)	Version B		Valence M (SD)	Arousal M (SD)	Version C		Valence M (SD)	Arousal M (SD)
**Negative**	1006	Savage attack from cannibal tribe to one anthropologist, dismembering him. Children start to eat parts of him	1.98 (1.50)	7.37 (1.88)	1002	Vinnie Jones removing the eyes and the teeth of the victim	1.67 (1.36)	7.72 (1.67)	1000	Amputation scene on top of the ruins	2.04 (1.98)	7.11 (1.77)
	1007	Jordana Brewster very scared, hidden on a box assists to a mutilation of her boyfriend	1.81 (1.43)	7.33 (1.91)	1005	Leslie Bibb inside a carriage with bodies hanging from the ceiling	2.06 (1.48)	6.92 (1.74)	1001	Leatherface removing the face of Mathew Bomer	1.68 (1.45)	7.45 (1.77)
	1009	Vinnie Jones with a vicious attack direct to a woman that ends with the decapitation of the victim	1.83 (1.24)	6.88 (1.70)	1008	Jaimie Alexander giving a merciful shot to the head of a police	1.94 (1.41)	6.53 (2.05)	1003	Jay Hernandez is being tortured on a chair and fingers form his hand are amputated	2.99 (2.00)	6.19 (2.20)
	3002	Emilia Fox brings a cloth to Adrien Brody that is lying sick on the bed	2.49 (1.45)	3.87 (2.15)	3000	Barry Watson entering a church for his mom's funeral	3.13 (1.62)	3.64 (2.14)	1004	Cannibalism scene	2.07 (1.91)	6.88 (1.95)
	3005	Danielle Savre crying in the arms of Matt Cohen	3.54 (1.53)	4.05 (1.96)	3001	Girl crying while her beloved died in her arms	3.04 (1.67)	4.79 (1.42)	3003	Very sad Belen Fabra considering suicide	2.65 (1.42)	4.29 (2.02)

Continued on next page

Categories	Version A		Valence M (SD)	Arousal M (SD)	Version B		Valence M (SD)	Arousal M (SD)	Version C		Valence M (SD)	Arousal M (SD)
**Negative**	3006	Josh Hutcherson crying in the arms of Robert Pat rick	2.88 (1.36)	4.25 (2.12)	3004	Marcia Gay Harden and Tim Robbins having an argument	2.88 (1.27)	3.79 (1.80)	3007	Annete Benning and Thora Birch having an argument, in Thora's room	3.20 (1.19)	3.58 (1.84)
	3008	Wes Bentley and Chris Cooper having an argument	3.33 (1.28)	3.61 (1.69)	7001	Teenage boy attacked by a group of young people in the toilet at the school	1.95 (1.37)	6.48 (2.30)	3009	Sean Penn crying in the front porch	3.30 (1.46)	3.89 (2.05)
	7007	Young woman filmed while being assaulted in a house	2.05 (1.27)	6.13 (2.33)	7004	Little boy attacked by a group of older children and dragged into the dustbin on the street	2.29 (1.32)	5.79 (2.35)	7000	Teenage boy abused by a group of young people on the street	1.92 (1.15)	6.21 (2.23)
	7008	Teenage boy attacked in the sports court	2.37 (1.48)	5.70 (2.25)	7005	Schoolmates bully a little boy	2.29 (1.39)	5.80 (2.37)	7002	Teenage girl beaten up in the classroom by other classmates	2.18 (1.27)	5.94 (2.38)
	7009	Homeless humiliated on the street	2.00 (1.29)	6.31 (2.29)	7006	Teenage boy assaulted in the school hallway by a group of students	2.25 (1.44)	5.54 (2.21)	7003	Teenage boy humiliated in locker room	2.18 (1.27)	5.85 (2.43)

Continued on next page

Categories	Version A		Valence M (SD)	Arousal M (SD)	Version B		Valence M (SD)	Arousal M (SD)	Version C		Valence M (SD)	Arousal M (SD)
**Neutral**	5005 (2x)	Sandstorm and desert	5.32 (1.06)	2.86 (1.97)	5002 (2x)	Scenery with polar scenes and the dusk	5.68 (1.70)	2.51 (1.86)	5001 (2x)	Mountains with ice	5.83 (1.67)	2.72 (2.03)
	6009 (2x)	Moving small, coloured paper triangles	4.98 (0.73)	2.14 (1.68)	6010 (2x)	Handling a small wooden puppet	5.06 (0.91)	2.20 (1.73)	6013 (2x)	Moving coloured paper trapezoids	4.91 (0.94)	2.21 (1.97)
	6004	Moving coloured paper squares	4.90 (0.94)	2.01 (1.82)	6011	Gait in different shoes/sleepers	5.08 (1.02)	2.50 (1.84)	6005	Moving coloured cups	5.11 (1.00)	2.15 (1.84)
	6002	Moving big, coloured paper triangles	5.14 (0.75)	1.85 (1.51)	6003	Moving highlighters of different colours	4.89 (1.09)	2.39 (2.04)	6012 (2x)	Putting cutlery in a tray	5.13 (0.65)	1.97 (1.60)
	6006	Painting a leaf	5.33 (1.32)	2,66 (1.92)	6007	Solving three different mazes	5.19 (1.34)	2,63 (2.07)	6008	Bouncing ball slowly moving in the hall	5.19 (0.95)	2.15 (1.87)

Table S2. Bayes factors for analyses based on the condition effect.

Outcome measure	BF_{10}	Interpretation
**Physiological outcomes**		
Heart Rate	0.150	Strong evidence for H_0
Skin Conductance	0.176	Strong evidence for H_0
Respiratory Rate	0.051	Strong evidence for H_0
**Subjective ratings**		
Valence	0.098	Strong evidence for H_0
Arousal	0.046	Strong evidence for H_0
**PANAS – Affective states**		
Positive Affect	0.037	Strong evidence for H_0
Negative Affect	0.080	Strong evidence for H_0
**VAS – Side effects**		
Symptom 1 – Anxiety	0.050	Strong evidence for H_0
Symptom 2 – Sadness	0.322	Strong evidence for H_0
Symptom 3 – Itch	0.455	Strong evidence for H_0
Symptom 4 – Headache	0.064	Strong evidence for H_0
Symptom 5 – Other pain	0.046	Strong evidence for H_0
Symptom 6 – Tingling	0.067	Strong evidence for H_0
Symptom 7 – Tiredness	0.051	Strong evidence for H_0
Symptom 8 – Agitation	0.377	Strong evidence for H_0
Symptom 9 – Sleepiness	0.083	Strong evidence for H_0
Symptom 10 – Metallic taste	0.106	Strong evidence for H_0

Note: BF = Bayes factor; H_0 = null hypothesis (no stimulation effect).

Table S3. Descriptive statistics for valence and arousal by stimulation condition and film category.

Conditions	**Valence**		**Arousal**	
	Neutral Films M (SD)	Negative Films M (SD)	Neutral Films M (SD)	Negative Films M (SD)
Sham	5.05 (0.44)	2.99 (0.73)	1.61 (1.23)	4.39 (1.79)
In-Phase	5.15 (0.50)	3.08 (0.78)	1.50 (.90)	4.37 (1.80)
Anti-Phase	5.05 (0.40)	3.10 (0.71)	1.60 (1.10)	4.32 (1.74)

Table S4. Random effects and model fit statistics for valence and arousal models.

Parameter	**Valence**	**Arousal**
**Random Effects (Variance Components)**		
Participant intercept (τ^2_{00})	0.068	1.028
Residual (σ^2)	0.306	1.137
**Variance Partitioning**		
ICC (Intraclass Correlation)	0.181	0.475
**Model Fit**		
Marginal R^2	0.735	0.475
Conditional R^2	0.783	0.724

Note: Random effects variance components: τ^2_{00} = between-participant variance in intercepts; σ^2 = within-participant residual variance. ICC = proportion of total variance attributable to between-participant differences. Marginal R^2 = variance explained by fixed effects only; Conditional R^2 = variance explained by both fixed and random effects.

Table S5. Fixed effects for linear mixed model predicting valence and arousal ratings.

	β	SE	95% CI	t	p
**Valence**					
Intercept	5.05	0.09	4.87, 5.23	53.56	p < 0.001
Condition (In-Phase vs. Sham)	0.10	0.12	−0.13, 0.34	0.86	0.389
Condition (Anti-Phase vs. Sham)	0.00	0.12	−0.24, 0.24	0.00	1.00
Film Category	−2.06	0.12	−2.29, −1.82	−17.05	p < 0.001
In-Phase × Film Category	−0.01	0.17	−0.35, 0.32	−0.08	0.936
Anti-Phase × Film Category	0.10	0.17	−0.23, 0.44	0.61	0.540
**Arousal**					
Intercept	1.61	0.23	1.16, 2.06	7.09	p < 0.001
Condition (In-Phase vs. Sham)	−0.11	0.23	−0.57, 0.35	−0.47	0.637
Condition (Anti-Phase vs. Sham)	−0.01	0.23	−0.47, 0.45	−0.04	0.969
Film Category (Negative vs. Neutral)	2.78	0.23	2.33, 3.24	11.96	p < 0.001
In-Phase × Film Category	0.09	0.33	−0.56, 0.74	0.27	0.788
Anti-Phase × Film Category	−0.06	0.33	−0.71, 0.58	−0.20	0.844

Note: N = 42 participants, 252 observations. df = 205 for all fixed effects (Satterthwaite approximation). Reference categories: Sham – Neutral. CI = confidence interval.

Description S2 – *Emotions*

Overall, the emotions selected by participants in reaction to the negative film clips were consistent across groups. In the sham group, the most frequently selected emotion was anger (19.52%), followed by aversion (16.19%). In contrast, participants in both active stimulation groups most frequently reported aversion (20.24% in each group) as their primary emotional feeling. The second most frequently selected emotion in active groups was anger (18.81% in the in-phase group and 17.62% in the anti-phase group). Sadness was the third most selected emotion in the sham and anti-phase groups

(15.71%), whereas in the in-phase group, the third most selected response was a neutral emotion (16.67%) (Table S5).

Table S6. Percentage of reported emotions by film category (Neutral vs. Negative).

Emotions	Sham		In-phase		Anti-phase	
	Neutral %	Negative %	Neutral %	Negative %	Neutral %	Negative %
Happiness	3.1	-	1.67	0.48	1.67	-
Sadness	1.43	15.71	1.19	14.05	-	15.71
Anger	1.67	19.52	1.43	18.81	1.90	17.62
Fear	0.95	10.24	0.71	6.43	0.95	6.43
Aversion	-	16.19	0.24	20.24	0.24	20.24
Surprise	1.19	3.10	0.95	2.86	0.95	5.24
Neutral	70.95	14.52	72.86	16.67	74.05	14.76
Solidarity	0.24	3.81	-	5.71	-	5.48
Compassion	-	8.57	0.24	7.62	-	7.62
Envy	-	-	-	-	-	-
Love	-	0.95	-	0.48	-	0.48
Longing	-	0.24	0.71	0.24	-	0.24
Blame	0.48	0.24	-	-	0.24	-
Pity	-	5.24	-	5.00	-	5.24
Shame	-	1.43	-	0.71	-	0.48
Jealousy	-	-	-	-	-	-
Embarrassment	-	0.24	-	0.71	-	0.48

Table S7. Descriptive statistics for physiological measures by stimulation condition, film category and time interval.

Measure	Condition	Film	**Total** M (SD)	**0–5 s** M (SD)	**5–10 s** M (SD)	**10–15 s** M (SD)	**15–20 s** M (SD)	**20–25 s** M (SD)	**25–30 s** M (SD)
Heart Rate	Sham	Neutral	75.39 (9.94)	75.90 (10.75)	76.07 (9.95)	75.40 (9.51)	75.07 (9.87)	75.07 (9.61)	74.85 (10.45)
	Sham	Negative	74.94 (9.95)	76.24 (10.30)	75.82 (10.24)	74.99 (9.86)	74.36 (9.80)	74.48 (9.68)	73.75 (10.15)
	In-Phase	Neutral	75.14 (9.47)	76.22 (9.73)	75.40 (9.60)	75.38 (9.44)	75.04 (9.47)	74.46 (9.29)	74.34 (9.71)
	In-Phase	Negative	74.74 (9.49)	75.20 (9.65)	75.37 (9.79)	74.81 (9.60)	74.80 (9.43)	74.41 (9.54)	73.83 (9.44)
	Anti-Phase	Neutral	76.24 (10.20)	77.15 (10.18)	76.73 (10.45)	76.51 (10.01)	75.78 (10.70)	75.91 (9.97)	75.36 (10.36)
	Anti-Phase	Negative	75.94 (9.84)	77.03 (10.08)	76.58 (9.83)	76.18 (9.78)	75.82 (9.52)	75.53 (9.89)	74.50 (10.32)
Skin Conductance	Sham	Neutral	−0.04 (0.08)	−0.03 (0.12)	−0.06 (0.10)	−0.03 (0.07)	−0.04 (0.05)	−0.04 (0.07)	−0.03 (0.06)
	Sham	Negative	−0.02 (0.11)	0.02 (0.16)	−0.03 (0.14)	−0.04 (0.06)	−0.01 (0.08)	−0.05 (0.09)	−0.02 (0.08)
	In-Phase	Neutral	−0.04 (0.09)	−0.05 (0.13)	−0.04 (0.13)	−0.05 (0.08)	−0.04 (0.05)	−0.03 (0.10)	−0.02 (0.06)
	In-Phase	Negative	−0.02 (0.10)	0.03 (0.15)	−0.03 (0.10)	−0.02 (0.08)	−0.03 (0.08)	−0.00 (0.10)	−0.04 (0.07)
	Anti-Phase	Neutral	−0.03 (0.06)	−0.03 (0.09)	−0.05 (0.06)	−0.02 (0.05)	−0.02 (0.06)	−0.04 (0.04)	−0.02 (0.05)
	Anti-Phase	Negative	−0.02 (0.09)	−0.02 (0.11)	−0.01 (0.10)	−0.01 (0.07)	−0.01 (0.07)	−0.02 (0.10)	−0.03 (0.05)
Respiratory Rate	Sham	Neutral	16.72 (2.93)	16.38 (2.90)	16.47 (2.91)	16.62 (2.90)	16.70 (3.15)	16.94 (2.99)	17.17 (2.82)
	Sham	Negative	17.56 (3.01)	17.27 (3.46)	17.57 (2.99)	17.95 (3.32)	17.47 (2.83)	17.45 (2.76)	17.64 (2.78)
	In-Phase	Neutral	16.21 (2.81)	16.30 (2.86)	15.98 (2.73)	16.05 (2.86)	16.20 (2.80)	16.47 (3.04)	16.28 (2.68)
	In-Phase	Negative	17.10 (2.85)	16.67 (2.83)	17.00 (2.85)	17.14 (3.05)	17.08 (2.82)	17.57 (2.96)	17.16 (2.72)
	Anti-Phase	Neutral	16.59 (3.03)	16.45 (3.12)	16.16 (3.03)	16.60 (3.11)	16.78 (3.31)	16.84 (3.11)	16.70 (2.61)
	Anti-Phase	Negative	17.16 (2.83)	16.78 (2.75)	17.20 (2.93)	17.23 (3.19)	17.26 (2.74)	17.20 (2.79)	17.31 (2.69)

Note: Values represent mean scores during negative and neutral film viewing. Heart Rate measured in beats per minute (bpm), Skin Conductance in microsiemens (µS), Respiratory Rate in breaths per minute (bpm). N = 42 participants per cell. This table shows temporal patterns across the 30-second film viewing period for each combination of measure, valence, and stimulation condition

Table S8. Descriptive statistics for physiological measures by stimulation condition and time interval.

Measure	Condition	**Total** M (SD)	**0–5 s** M (SD)	**5–10 s** M (SD)	**10–15 s** M (SD)	**15–20 s** M (SD)	**20–25 s** M (SD)	**25–30 s** M (SD)
Heart Rate	Sham	−0.46 (3.72)	0.34 (2.93)	−0.25 (3.60)	−0.42 (4.03)	−0.72 (4.82)	−0.59 (3.14)	−1.10 (3.79)
	In-Phase	−0.40 (3.33)	−1.02 (4.88)	−0.03 (3.12)	−0.57 (3.11)	−0.23 (2.97)	−0.05 (2.76)	−0.51 (3.18)
	Anti-Phase	−0.30 (2.86)	−0.13 (3.43)	−0.15 (2.67)	−0.34 (2.67)	0.04 (3.09)	−0.37 (2.32)	−0.86 (3.00)
Skin Conductance	Sham	0.02 (0.10)	0.06 (0.14)	0.03 (0.14)	−0.01 (0.07)	0.03 (0.09)	−0.01 (0.09)	0.01 (0.08)
	In-Phase	0.02 (0.12)	0.08 (0.13)	0.01 (0.13)	0.03 (0.11)	0.00 (0.09)	0.03 (0.18)	−0.01 (0.09)
	Anti-Phase	0.01 (0.10)	0.01 (0.14)	0.04 (0.12)	0.01 (0.09)	0.00 (0.09)	0.02 (0.11)	−0.01 (0.08)
Respiratory Rate	Sham	0.80 (1.96)	0.63 (1.51)	1.10 (1.79)	1.33 (3.04)	0.77 (1.92)	0.51 (1.74)	0.47 (1.76)
	In-Phase	0.86 (2.43)	0.38 (1.84)	1.02 (1.95)	1.08 (2.11)	0.84 (1.84)	1.10 (1.89)	0.88 (1.67)
	Anti-Phase	0.87 (2.38)	0.46 (1.69)	1.23 (1.91)	0.78 (2.62)	0.71 (2.14)	0.65 (1.97)	0.74 (2.21)

Note: Values represent mean difference between negative and neutral films (negative – neutral). Heart Rate measured in beats per minute (bpm), Skin Conductance in microsiemens (μS), Respiratory Rate in breaths per minute (bpm). N = 42 participants per cell. This table shows temporal patterns across the 30-second film viewing period for each combination of measure, and stimulation condition.

Table S9. Random effects and model fit statistics for physiological measures.

Parameter	**Heart Rate**	**Skin Conductance**	**Respiratory Rate**
**Random Effects (Variance Components)**			
Participant intercept (τ^2_{00})	0.09	0.00	1.18
Participant: Condition (τ^2_{01})	4.46	0.00	0.79
Residual (σ^2)	6.83	0.01	2.10
**Variance Partitioning**			
ICC (Intraclass Correlation)	0.588	0.468	0.521
**Model Fit**			
Marginal R^2	0.044	0.022	0.056
Conditional R^2	0.606	0.480	0.548

Note: Random effects variance components: τ^2_{00} = between-participant variance in intercepts; τ^2_{01} = variance in participant responses across stimulation conditions; σ^2 = within-participant residual variance. ICC = proportion of total variance attributable to participant-level differences (both random effects combined). Marginal R^2 = variance explained by fixed effects only; Conditional R^2 = variance explained by both fixed and random effects. All models included Film Category × Stimulation.

Table S10. Fixed effects for linear mixed model predicting heart rate.

Predictor	β	SE	95% CI	t	p
Intercept	−0.46	0.37	−1.19, 0.28	−1.24	0.218
Condition (In-Phase vs. Sham)	0.05	0.52	−0.97, 1.07	0.10	0.919
Condition (Anti-Phase vs. Sham)	0.15	0.52	−0.86, 1.17	0.30	0.766
Time Interval (Linear)	−1.01	0.40	−1.80, −0.22	−2.52	0.012
In-Phase × Time (Linear)	1.35	0.57	0.23, 2.47	2.37	0.018
Anti-Phase × Time (Linear)	0.54	0.57	−0.58, 1.66	0.95	0.342

Note: N = 42 participants, 756 observations (3 conditions × 6 time intervals). df = 615 for time intervals and interactions; df = 82 for stimulation conditions; df = 123 for intercept (Satterthwaite approximation). Reference category: Sham condition.

Table S11. Fixed effects for linear mixed model predicting skin conductance.

Predictor	β	SE	95% CI	t	p
Intercept	0.01	0.01	0.00, 0.03	2.30	0.022
Condition (In-Phase vs. Sham)	0.01	0.01	−0.01, 0.03	0.63	0.531
Condition (Anti-Phase vs. Sham)	−0.01	0.01	−0.03, 0.02	−0.54	0.590
Time Interval (Linear)	−0.04	0.02	−0.07, −0.00	−2.16	0.031
In-Phase × Time (Linear)	−0.01	0.03	−0.06, 0.04	−0.46	0.643
Anti-Phase × Time (Linear)	0.02	0.03	−0.03, 0.07	0.81	0.418

Note: N = 42 participants, 756 observations (3 conditions × 6 time intervals). df = 615 for time intervals and interactions; df = 82 for stimulation conditions; df = 123 for intercept (Satterthwaite approximation). Reference category: Sham condition.

Table S12. Fixed effects for linear mixed model predicting respiratory rate.

Predictor	β	SE	95% CI	t	p
Intercept	0.79	0.23	0.33, 1.26	3.38	0.001
Condition (In-Phase vs. Sham)	0.07	0.23	−0.39, 0.54	0.31	0.756
Condition (Anti-Phase vs. Sham)	0.02	0.24	−0.45, 0.48	0.08	0.940
Time Interval (Linear)	−0.35	0.22	−0.79, 0.09	−1.55	0.121
In-Phase × Time (Linear)	−0.51	0.22	−0.95, −0.07	−2.27	0.024
Anti-Phase × Time (Linear)	0.65	0.32	0.02, 1.28	2.03	0.042

Note: N = 42 participants, 734 observations (3 conditions × 6 time intervals). df = 596 for time intervals and interactions; df = 80 for stimulation conditions; df = 80 for intercept (Satterthwaite approximation). Time intervals coded using orthogonal polynomial contrasts. Reference category: Sham condition.

Table S13. Descriptive statistics for PANAS scores by affect type, stimulation condition, and time.

Affect Type	Condition	Time	M	SD
Positive Affect	Sham	Pre	28.1	7.80
	Sham	Post	25.8	8.76
	In-Phase	Pre	28.8	7.00
	In-Phase	Post	26.5	7.66
	Anti-Phase	Pre	28.7	7.54
	Anti-Phase	Post	25.6	7.66
Negative Affect	Sham	Pre	12.7	3.53
	Sham	Post	12.8	4.02
	In-Phase	Pre	13.0	3.64
	In-Phase	Post	12.2	2.54
	Anti-Phase	Pre	12.3	2.39
	Anti-Phase	Post	11.8	2.12

Note: PANAS = Positive and Negative Affect Schedule. Positive Affect scores range from 10–50; Negative Affect scores range from 10–50. N = 42 participants per cell. Pre = before film viewing; Post = after film viewing.

Table S14. Random effects and model fit statistics for PANAS models.

Parameter	**Positive Affect**	**Negative Affect**
**Random Effects (Variance Components)**		
Participant intercept (τ^2_{00})	42.05	1.91
Residual (σ^2)	18.11	7.84
**Variance Partitioning**		
ICC (Intraclass Correlation)	0.699	0.196
**Model Fit**		
Marginal R^2	0.029	0.017
Conditional R^2	0.708	0.210

Note: Random effects variance components: τ^2_{00} = between-participant variance in intercepts; σ^2 = within-participant residual variance. ICC = proportion of total variance attributable to between-participant differences. Marginal R^2 = variance explained by fixed effects only; Conditional R^2 = variance explained by both fixed and random effects. Both models included Condition × Time interactions with random intercepts for participants.

Table S15. Fixed effects for linear mixed model predicting positive affect scores.

Predictor	β	SE	95% CI	t	p
Intercept	28.14	1.20	25.79, 30.49	23.52	$p < 0.001$
Condition (In-Phase vs. Sham)	0.69	0.93	−1.14, 2.52	0.74	0.458
Condition (Anti-Phase vs. Sham)	0.60	0.93	−1.23, 2.43	0.64	0.522
Time (Post vs. Pre)	−2.31	0.93	−4.14, −0.48	−2.49	0.014
In-Phase × Time	−0.02	1.31	−2.61, 2.57	−0.02	0.986
Anti-Phase × Time	−0.86	1.31	−3.45, 1.73	−0.65	0.515

Note: N = 42 participants, 252 observations. df = 205 for Time and interactions; df = 71 for Intercept (Satterthwaite approximation). Reference categories: Sham – Pre.

Table S16. Fixed effects for linear mixed model predicting negative affect scores.

Predictor	β	SE	95% CI	t	p
	12.71	0.48	11.77, 13.66	26.38	$p < 0.001$
Condition (In-Phase vs. Sham)	0.29	0.61	−0.91, 1.49	0.47	0.641
Condition (Anti-Phase vs. Sham)	−0.38	0.61	−1.58, 0.82	−0.62	0.534
Time (Post vs. Pre)	0.07	0.61	−1.13, 1.27	0.12	0.907
In-Phase × Time	−0.90	0.86	−2.60, 0.79	−1.05	0.296
Anti-Phase × Time	−0.60	0.86	−2.30, 1.11	−0.69	0.492

Note: N = 42 participants, 252 observations. df = 205 for all fixed effects (Satterthwaite approximation). Reference categories: Sham – Pre.

Table S17. Descriptive statistics for vas scores by side effects, stimulation condition, and time.

		**Tiredness**	**Anxiety**	**Sadness**	**Agitation**	**Sleepiness**
Condition	Time	M (SD)	M (SD)	M (SD)	M (SD)	M (SD)
Sham	Pre	3.98 (2.26)	2.38 (2.42)	0.57 (1.27)	1.88 (1.98)	3.45 (2.68)
Sham	Post	4.02 (2.44)	1.62 (2.00)	0.69 (1.33)	1.67 (2.23)	4.17 (2.70)
In-phase	Pre	3.93 (2.79)	2.64 (2.62)	0.67 (1.34)	1.95 (2.31)	3.83 (3.01)
In-phase	Post	3.98 (3.07)	1.67 (1.95)	0.50 (1.31)	1.90 (2.61)	3.93 (3.12)
Anti-phase	Pre	4.10 (2.39)	2.02 (2.07)	0.36 (0.73)	1.86 (2.13)	3.83 (3.05)
Anti-phase	Post	4.10 (2.58)	0.95 (1.87)	0.21 (0.65)	1.60 (2.00)	4.12 (3.01)
		**Itch**	**Headache**	**Another pain**	**Tingling**	**Metallic taste**
Condition	Time	M (SD)	M (SD)	M (SD)	M (SD)	M (SD)
Sham	Pre	0.12 (0.50)	0.67 (1.43)	0.26 (1.11)	0.00 (0.00)	0.02 (0.15)
Sham	Post	0.24 (0.69)	1.07 (1.99)	0.10 (0.48)	0.12 (0.50)	0.02 (0.15)
In-phase	Pre	0.07 (0.34)	0.52 (1.13)	0.26 (0.94)	0.02 (0.15)	0.00 (0.00)
In-phase	Post	0.43 (1.27)	1.07 (1.96)	0.31 (1.00)	0.10 (0.37)	0.02 (0.15)
Anti-phase	Pre	0.17 (0.44)	0.60 (1.42)	0.05 (0.31)	0.00 (0.00)	0.00 (0.00)
Anti-phase	Post	0.45 (1.47)	0.88 (1.63)	0.00 (0.00)	0.05 (0.22)	0.00 (0.00)

Table S18. Random effects and model fit statistics for linear mixed models.

	**Random Effects (Variance Components)**		**Variance Partitioning**	**Model Fit**	
**Side Effect**	Participant intercept (τ^2_{00})	Residual (σ^2)	ICC (Intraclass Correlation)	Marginal R^2	Conditional R^2
Tiredness	2.966	3.810	0.438	0.001	0.438
Anxiety	1.801	2.913	0.382	0.061	0.420
Sadness	0.608	1.246	0.330	0.080	0.393
Agitation	1.288	2.995	0.300	0.009	0.306
Sleepiness	4.883	3.786	0.563	0.004	0.566
Itch	0.149	0.576	0.205	0.027	0.226
Headache	0.866	2.022	0.300	0.013	0.309
Another pain	0.196	0.407	0.325	0.080	0.379
Tingling	0.003	0.005	0.400	0.026	0.407
Metallic taste	0.005	0.007	0.400	0.012	0.407

Note: Random effects variance components: τ^2_{00} = between-participant variance in intercepts; σ^2 = within-participant residual variance. ICC = proportion of total variance attributable to between-participant differences. Marginal R^2 = variance explained by fixed effects only; Conditional R^2 = variance explained by both fixed and random effects.

Table S19. Fixed effects for linear mixed model predicting side effects.

	β	SE	95% CI	t	p
**Tiredness**					
Intercept	3.98	0.40	3.18, 4.78	9.90	p < 0.001
Condition (In-phase vs. Sham)	−0.05	0.43	−0.89, 0.79	−0.11	0.911
Condition (Anti-phase vs. Sham)	0.12	0.43	−0.72, 0.96	0.28	0.780
Time (Post vs. Pre)	0.05	0.43	−0.79, 0.89	0.11	0.911
In-phase × Time	0.00	0.60	−1.18, 1.18	0.00	1.00
Anti-phase × Time	−0.05	0.60	−1.23, 1.13	−0.08	0.937
**Anxiety**					
Intercept	2.38	0.34	1.72, 3.04	7.11	p < 0.001
Condition (In-phase vs. Sham)	0.26	0.37	−0.47, 0.99	0.70	0.483
Condition (Anti-phase vs. Sham)	−0.36	0.37	−1.09, 0.37	−0.96	0.339
Time (Post vs. Pre)	−0.76	0.37	−1.49, −0.03	−2.05	0.042
In-phase × Time	−0.21	0.53	−1.25, 0.83	−0.41	0.685
Anti-phase × Time	−0.31	0.53	−1.35, 0.73	−0.59	0.557
**Sadness**					
Intercept	0.57	0.18	0.22, 0.92	3.24	0.002
Condition (In-phase vs. Sham)	0.10	0.19	−0.28, 0.47	0.50	0.616
Condition (Anti-phase vs. Sham)	−0.21	0.19	−0.59, 0.16	−1.13	0.260
Time (Post vs. Pre)	0.12	0.19	−0.25, 0.49	0.63	0.531
In-phase × Time	−0.29	0.27	−0.81, 0.24	−1.06	0.288
Anti-phase × Time	−0.26	0.27	−0.79, 0.27	−0.98	0.330
**Agitation**					
Intercept	1.88	0.34	1.21, 2.55	5.50	p < 0.001
Condition (In-phase vs. Sham)	0.07	0.33	−0.57, 0.71	0.22	0.827
Condition (Anti-phase vs. Sham)	−0.02	0.33	−0.66, 0.62	−0.07	0.942
Time (Post vs. Pre)	−0.21	0.33	−0.86, 0.43	−0.66	0.513
In-phase × Time	0.17	0.46	−0.74, 1.08	0.36	0.719
Anti-phase × Time	−0.05	0.46	−0.96, 0.86	−0.10	0.918
**Sleepiness**					
Intercept	3.45	0.45	2.56, 4.34	7.62	p < 0.001
Condition (In-phase vs. Sham)	0.38	0.43	−0.47, 1.23	0.88	0.379
Condition (Anti-phase vs. Sham)	0.38	0.43	−0.47, 1.23	0.88	0.379
Time (Post vs. Pre)	0.71	0.43	−0.14, 1.56	1.65	0.100
In-phase × Time	−0.62	0.61	−1.82, 0.58	−1.01	0.312
Anti-phase × Time	−0.43	0.61	−1.63, 0.77	−0.70	0.484
**Itch**					
Intercept	0.12	0.14	−0.15, 0.39	0.86	0.390
Condition (In-phase vs. Sham)	−0.05	0.19	−0.42, 0.32	−0.25	0.803
Condition (Anti-phase vs. Sham)	0.05	0.19	−0.32, 0.42	0.25	0.803
Time (Post vs. Pre)	0.12	0.19	−0.26, 0.49	0.62	0.533
In-phase × Time	0.24	0.27	−0.29, 0.77	0.88	0.378

Continued on next page

	β	SE	95% CI	t	p
Anti-phase × Time	0.17	0.27	−0.36, 0.70	0.62	0.537
**Headache**					
Intercept	0.67	0.25	0.18, 1.16	2.67	0.009
Condition (In-phase vs. Sham)	−0.14	0.26	−0.66, 0.37	−0.55	0.586
Condition (Anti-phase vs. Sham)	−0.07	0.26	−0.59, 0.45	−0.27	0.785
Time (Post vs. Pre)	0.40	0.26	−0.11, 0.92	1.55	0.123
In-phase × Time	0.14	0.37	−0.59, 0.87	0.39	0.700
Anti-phase × Time	−0.12	0.37	−0.85, 0.61	−0.32	0.748
**Another pain**					
Intercept	0.26	0.12	0.03, 0.49	2.24	0.026
Condition (In-phase vs. Sham)	0.00	0.13	−0.26, 0.26	0.00	1.00
Condition (Anti-phase vs. Sham)	−0.21	0.13	−0.48, 0.05	−1.62	0.107
Time (Post vs. Pre)	−0.17	0.13	−0.43, 0.09	−1.26	0.209
In-phase × Time	0.21	0.19	−0.16, 0.59	1.15	0.253
Anti-phase × Time	0.12	0.19	−0.25, 0.49	0.64	0.525
**Tingling**					
Intercept	<0.001	0.04	−0.08, 0.08	0.00	1.00
Condition (In-phase vs. Sham)	0.02	0.06	−0.09, 0.14	0.40	0.689
Condition (Anti-phase vs. Sham)	<0.001	0.06	−0.12, 0.12	0.00	1.00
Time (Post vs. Pre)	0.12	0.06	0.00, 0.24	2.01	0.046
In-phase × Time	−0.05	0.08	−0.21, 0.12	−0.57	0.571
Anti-phase × Time	−0.07	0.08	−0.24, 0.09	−0.85	0.395
**Metallic taste**					
Intercept	0.02	0.02	−0.01, 0.06	1.41	0.160
Condition (In-phase vs. Sham)	−0.02	0.02	−0.06, 0.01	−1.29	0.198
Condition (Anti-phase vs. Sham)	−0.02	0.02	−0.06, 0.01	−1.29	0.198
Time (Post vs. Pre)	<0.001	0.02	−0.04, 0.04	0.00	1.00
In-phase × Time	0.02	0.03	−0.03, 0.08	0.91	0.362
Anti-phase × Time	<0.001	0.03	−0.05, 0.05	0.00	1.00

Note: N = 42 participants, 252 observations per side effect analysis. df = 205 for Time and interactions; df varies by side effect for Intercept (Satterthwaite approximation). Reference categories: Sham – Pre.

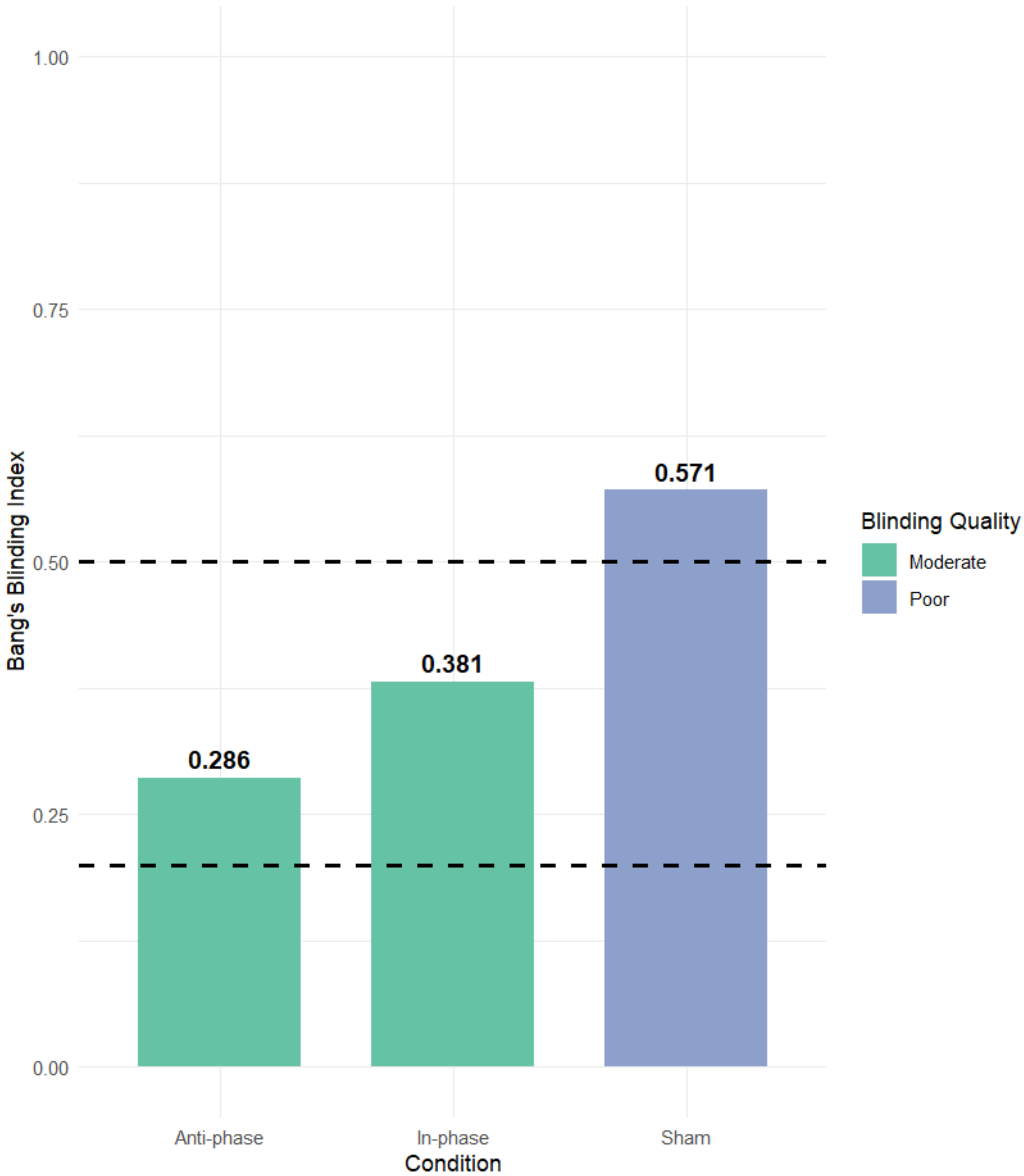

Figure S1. Bang's blinding index by condition. Note: Responses of 'I don't know' were classified as incorrect responses (sham and in-phase 6 participants; anti-phase 7 participants); dashed lines show thresholds: 0.2 (Good) and 0.5 (Poor).

AIMS Press

© 2026 the Author(s), licensee AIMS Press. This is an open access article distributed under the terms of the Creative Commons Attribution License (https://creativecommons.org/licenses/by/4.0)
